# Inhibition of UTX/KDM6A improves recovery of spinal cord injury by attenuating BSCB permeability and macrophage infiltration through the MLCK/p-MLC pathway

**DOI:** 10.1186/s12974-023-02936-1

**Published:** 2023-11-11

**Authors:** Yong Xie, Zixiang Luo, Wei Peng, Yudong Liu, Feifei Yuan, Jiaqi Xu, Yi Sun, Hongbin Lu, Tianding Wu, Liyuan Jiang, Jianzhong Hu

**Affiliations:** 1 Department of Spine Surgery and Orthopaedics, Xiangya Hospital, Central South University, Changsha, China; 2 Department of Sports Medicine, Xiangya Hospital, Central South University, Changsha, China; 3 Key Laboratory of Organ Injury, Aging and Regenerative Medicine of Hunan Province, Changsha, China; 4 Hunan Engineering Research Center of Sports and Health, Changsha, China; 5 National Clinical Research Center for Geriatric Disorders, Xiangya Hospital, Central South University, Changsha, China

**Keywords:** Spinal cord injury, Blood–spinal cord barrier, UTX/KDM6A, MYLK/MLCK

## Abstract

Spinal cord injury (SCI) can prompt an immediate disruption to the blood–spinal cord barrier (BSCB). Restoring the integrity of this barrier is vital for the recovery of neurological function post-SCI. The UTX protein, a histone demethylase, has been shown in previous research to promote vascular regeneration and neurological recovery in mice with SCI. However, it is unclear whether UTX knockout could facilitate the recovery of the BSCB by reducing its permeability. In this study, we systematically studied BSCB disruption and permeability at different time points after SCI and found that conditional UTX deletion in endothelial cells (ECs) can reduce BSCB permeability, decrease inflammatory cell infiltration and ROS production, and improve neurological function recovery after SCI. Subsequently, we used RNA sequencing and ChIP-qPCR to confirm that conditional UTX knockout in ECs can down-regulate expression of myosin light chain kinase (MLCK), which specifically mediates myosin light chain (MLC) phosphorylation and is involved in actin contraction, cell retraction, and tight junctions (TJs) protein integrity. Moreover, we found that MLCK overexpression can increase the ratio of p-MLC/MLC, further break TJs, and exacerbate BSCB deterioration. Overall, our findings indicate that UTX knockout could inhibit the MLCK/p-MLC pathway, resulting in decreased BSCB permeability, and ultimately promoting neurological recovery in mice. These results suggest that UTX is a promising new target for treating SCI.

**Graphical Abstract:**

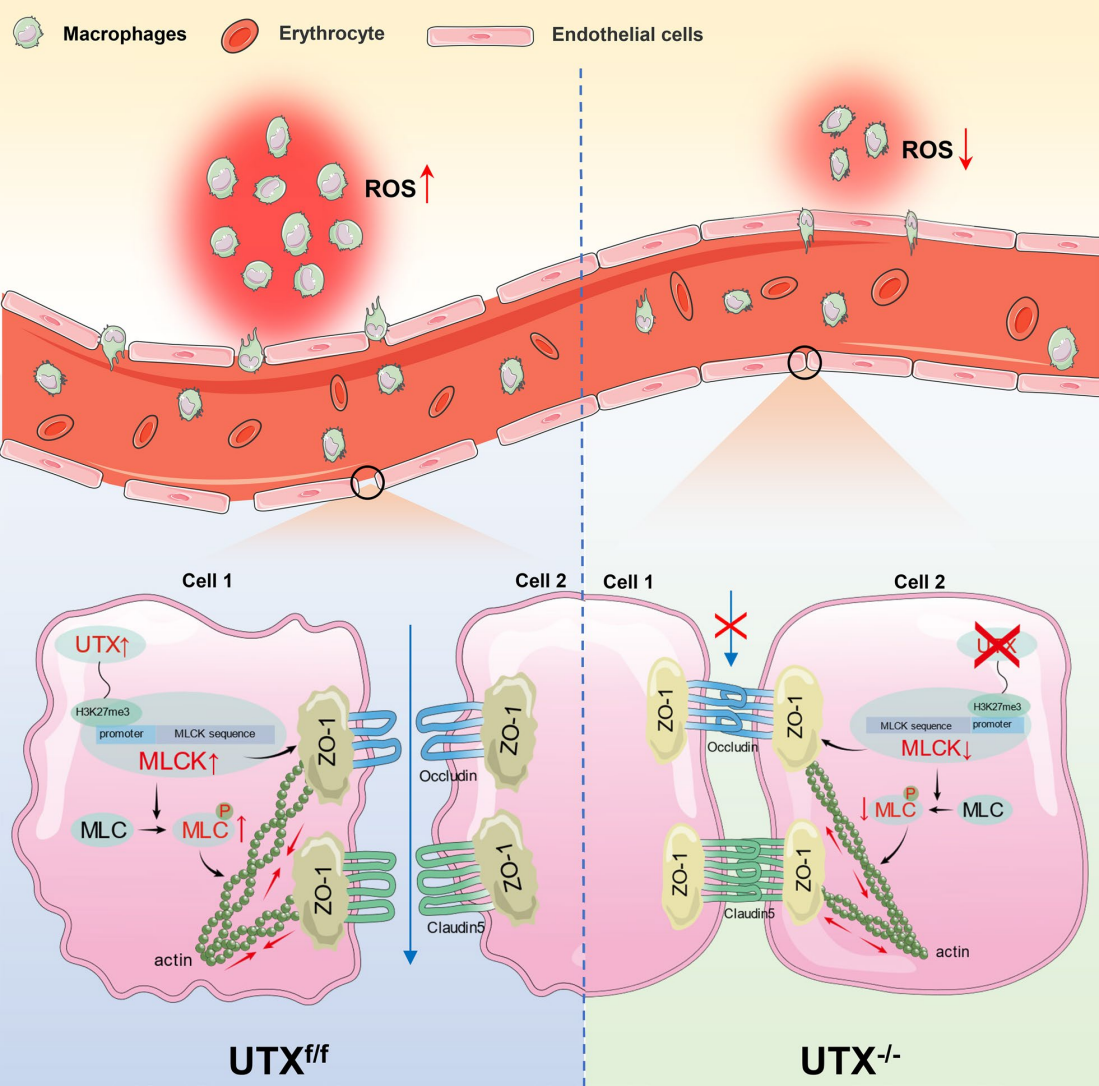

**Supplementary Information:**

The online version contains supplementary material available at 10.1186/s12974-023-02936-1.

## Introduction

Spinal cord injury (SCI) represents a severe neurological condition associated with long-term functional impairments. Globally, an estimated 27 million individuals grapple with the long-term disabilities stemming from SCI, with roughly 930,000 new incidences reported annually [1, 2]. The impact of SCI can be profound and can affect various aspects of a patient's life, including mobility, bowel and bladder function, and chronic pain. However, recovering neurological function after SCI remains a major challenge due to the adverse alteration of the injured microenvironment, which hampers axonal regeneration and meaningful reconnection [3, 4].

The blood–spinal cord barrier (BSCB) is essential for maintaining the normal function of the spinal cord, which prevents the entry of detrimental substances and toxins into the spinal cord tissue [5–7]. Primary injury to the spinal cord can cause disruption of the BSCB, resulting in augmented vascular permeability and subsequent leakage of blood components into the spinal cord tissue. This perturbation can trigger a succession of pathological events, potentially culminating in secondary SCI and further exacerbating neurological function decline [8]. It should be noted that neovascularization commences around 3–4 days after SCI and typically peaks around day 7 [9, 10]. Moreover, the newly formed blood vessels tend to exhibit dilated, tortuous morphologies and unusually high permeability [11]. And, the aberrant vascularization post-SCI can lead to the infiltration of peripheral macrophages, leukocytes, neutrophils into the spinal cord tissue, which can release inflammatory mediators, including reactive oxygen species (ROS) and further exacerbate the injured microenvironment and cause neuronal death [12]. The cascading pathological reactions, stemming from BSCB disruption, are intricate and wield enduring consequences on the injured milieu, thereby complicating neuronal remodeling efforts. The mechanism that regulates the integrity of the BSCB following SCI is not yet fully understood. Therefore, investigating the molecular mechanisms that uphold the integrity of the BSCB and facilitate remodeling of the BSCB post-SCI may unveil prospective therapeutic targets.

Epigenetic modulations significantly influence a spectrum of pathological disorders within the central nervous system [13]. In neurological diseases, epigenetic mechanisms regulating vascular permeability have also been reported in different diseases [14–17]. UTX, also known as KDM6A, functions as a pivotal demethylase, orchestrating an array of processes spanning neurological, oncological, and hematological disorders. Furthermore, it is instrumental in stem cell differentiation and embryonic development through the specific demethylation of H3 lysine-27 tri/dimethylation (H3K27me3/2) [18–24]. In a preceding study, we discerned a heightened expression of UTX in endothelial cells (ECs) post-SCI, culminating at a zenith on day 7. Subsequently, we conducted a study on mice and found that conditional ablation of UTX in ECs fostered enhanced vascular regeneration and bolstered neurological recuperation post-SCI [25]. Yet, the capacity of UTX deletion to potentiate neovascular stabilization, and by extension, ameliorate vascular permeability remains an enigma. Additionally, the mechanistic underpinnings governing this phenomenon are yet to be delineated.

In the present study, we aim to investigate the effect of UTX ablation on the permeability of the BSCB following SCI. Our findings reveal that conditional ablation of UTX in ECs engenders a diminution in vascular permeability, coupled with enhanced neurological recovery. Further, we discerned that UTX ablation can impede the expression of myosin light-chain kinase (MLCK). MLCK is implicated in the phosphorylation of endothelial myosin light chain (MLC), a process which triggers actomyosin contractility, undermines endothelial TJs, and escalates BSCB permeability. Our observations intimate that UTX ablation attenuates the BSCB permeability by inhibiting the MLCK/p-MLC pathway, thus preserving the integrity of endothelial cell TJs. This study presents a novel theoretical framework for the management of SCI and the restoration of neurological function.

## Materials and methods

### Animals and surgical procedures

The Ethics Committee of the Central South University (CSU) for Scientific Research approved all procedures related to the breeding, care, and experimentation of animals. We acquired C57BL/6J (wild type, WT) mice from Charles River. UTX^flox/flox^ strain (stock no. 021926) was procured from Jackson Laboratory. The Tek-Cre (SJ-008863) strain was purchased from Shanghai Model Organisms. By interbreeding heterozygous Tek-Cre mice with homozygous UTX^flox/flox^ mice, we derived the following genotypes: WT, Tek-Cre, UTX^flox/flox^(UTX^f/f^), Tek-Cre; UTX^flox/flox^(UTX^−/−^). We selected female mice aged 8 to 12 weeks. After laminectomy exposed the spinal cord in the mice's 10th thoracic vertebra under severe anesthesia (ketamine/xylazine/acepromazine, 50: 5: 1 mg/kg, i.p.), we employed Allen's weight-drop device to build the SCI model. To expose the spinal cord without inducing SCI, only a laminectomy was performed for the sham model.

### Evans Blue dye assays

Upon establishment of the SCI model in mice, 0.1 ml of 2% Evans Blue dye (EB; Sigma Aldrich, USA) was administered via tail vein injection at predetermined intervals. One hour post-injection, the spinal cord tissue was removed after perfusion. In brief, once the mice were anesthetized, the thoracic cavity was opened, revealing the heart. The right auricle was then clipped, and the mice underwent transcardial perfusion with 0.9% saline. This was later substituted with 4% paraformaldehyde to maintain the perfusion process. Upon completing the perfusion, a segment of the spinal cord, approximately 1 cm from the injury's epicenter, was dissected. Initially, digital images were captured to assess the leakage of EB. Subsequently, the spinal cord tissue underwent quantitative fluorescence analysis using near-infrared fluorescence imaging (NIRF, Perkin Elmer, USA) at an excitation wavelength of 620 nm and an emission wavelength of 680 nm. The quantification was expressed in radiant efficiency units. Ultimately, sections were frozen and imaged using fluorescence microscopy (Zeiss Apotome 2, Germany). Quantitative analyses were conducted using ImageJ software.

### Transmission electron microscopy (TEM)

The spinal cord tissue samples were excised, then fixed in 2.5% glutaraldehyde for 6 h, followed by dehydration. The tissue was subsequently embedded in epoxy resin and ultra-thin sections were prepared for examination under a transmission electron microscope (TEM; Hitachi, JPN). Two independent, blinded examiners utilized Image J software to evaluate and average the length, integrity of TJs, and thickness of basement membranes (BMs) in line with established standards [26].

### Isolation, culture, and identification of spinal cord microvascular ECs (SCMECs)

Primary mouse SCMECs were isolated following the protocol outlined in earlier studies [27]. In brief, the mouse spinal cord was excised, sectioned, and then subjected to digestion using 0.1% (v/v) type II collagenase (Sigma, USA). Subsequently, 20 ml of BSA-DMEM (20%, w/v) was introduced, followed by the removal of the upper myelin sheath. The tissue was further digested using 0.1% (v/v) collagenase/dispase (Sigma, USA). Thereafter, equal volumes of the medium were neutralized and the mixture was subjected to centrifugation. The supernatant was discarded, and the pelleted cells were identified as SCMECs, and subsequently seeded uniformly in culture flasks for subsequent experiments.

### Oxygen glucose deprivation and reoxygenation (OGD/R)

SCMECs were subjected to OGD as previously described to mimic the in vivo ischemic–hypoxic environment [28]. In a brief, they were cultivated in a medium free of glucose(DMEM without glucose; Gibco, USA). Then put in a humidified anaerobic chamber (Don Whitley Scientific, UK) with a 5% CO2 and 95% N2 environment (the oxygen concentration was < 0.2%) and maintained at 37 °C for 6 h. After that, the cells were moved to standard growth conditions (DMEM + 10% FBS in normoxic conditions, Inner Mongolia Opcel Biotechnology, China) for the reoxygenation experiments for 24 h.

### In vitro trans-endothelial permeability assay

To determine the permeability of the trans-endothelial, we used Fluorescein isothiocyanate (FITC)-dextran. SCMECs were seeded into the upper chamber of a 24-well transwell chamber with a 0.4-μm filter insert (Corning, USA) and allowed to reach full confluence. After OGD treatment, 40-kDa FITC-dextran (1 mg/mL; Sigma, USA) was administered into the upper chamber. A Varioskan LUX Multifunctional Enzyme Labeler (Thermo Fisher Scientific, USA) was used to detect the fluorescence intensity at 490 nm and 520 nm after 40 μl of medium had been removed from the lower chamber and shaded for 1 h.

### Evaluation of the locomotive function

The Basso Mouse Scale (BMS)system was used to evaluate the motor function in the mouse hindlimbs [29]. The BMS score runs from 0 to 9 (a score of 0 indicated complete paralysis and a score of 9 indicated normal locomotion). The BMS subscore consists of 11 points, including plantar pedaling frequency, bilateral hindlimb coordination, paw position, trunk stability, and tail position, higher scores indicate better motor function. Each mouse was monitored for 5 min by two experienced researchers who were blind to the experimental design and familiar with the BMS scores, who then recorded the BMS score and sub-scores. We conducted a footprint analysis at the same time [30]. To collect their footprints, mice had their forelimbs and hindlimbs painted with blue and red dyes, respectively, and then walked across a small, white paper-paved track that was 80 cm long and 4 cm wide. The length of the step taken with the hind paws was referred to as the stride. The stride width was determined by measuring the space between the left and right outermost toes. On each side, the totals of all measurements were averaged after 3 straight steps.

### Neuroelectrophysiology

Motor-evoked potentials (MEPs) were assessed and electrodes were implanted as in the earlier study [31] following the successful anesthesia of the mice. In brief, the stimulating electrode was set to the surface of the skull corresponding to the cortical motor area. At the same time, the recording electrode was inserted into the anterior tibial muscle of the contralateral hind limb. The reference electrode was placed in the subcutaneous tissue between the stimulating and recording electrodes. Mean MEP values were measured in various intervention groups before surgery (baseline) and 56 days after SCI. The relative quantity of motor recovery is represented for each mouse by the experimental MEP (mV).

### Lentiviral transfection

The MLCK overexpression lentiviral vector was constructed by GENECHEM (Shanghai, China). Cells were grown uniformly in 6-well culture plates, and transfection experiments were performed when cells grew to approximately 60–80% confluency. In vivo transfection was performed by intrathecal injection of 1 µl of lentivirus using a stereotaxic instrument.

### RNA-seq

For RNA-Sequencing, The Aksomics Corporation constructed the library and performed the sequencing (Aksomics, China). Briefly, UTX^f/f^ SCMECs and UTX^−/−^ SCMECs total RNA were extracted using Trizol reagent (Invitrogen, USA). Total RNA samples were enriched by oligo dT and then KAPA Stranded RNA-Seq Library Prep Kit (Illumina, USA) was used to construct the library, followed by sequencing using an Illumina NovaSeq 6000 sequencer (Illumina, USA). Each group contains three biological replicates.

### ChIP-qPCR

ChIP-qPCR was used to determine if H3K27me3 could bind to the MLCK promoter. ChIP assays were performed using the SimpleChIP® Enzymatic Chromatin IP Kit (Magnetic Beads) #9003 (CST, USA). The antibodies used for ChIP were Tri-Methyl-Histone H3 antibody and Rabbit IgG antibody (CST, USA). Design of primers for targeting the MLCK promoter region: P1-Forward, 5′-GACCCTGTCCTGGTATGGC-3′; Reverse, 5′-GCACGCACTCGGAATTTGT-3′, P2-Forward, 5′-AAGAAATGGGCACGATGC-3′; Reverse, 5′-TGCCTGATGGGAATGCTA-3′, P3- Forward, 5′-GACGGAGCGGGAGTGTATC-3′; Reverse, 5′-ATGTTCTCGGGCTTGAGGT-3′. The ChIP-qPCR products were characterized by 2% agarose gel electrophoresis. Values from ChIP-qPCR were normalized to those of the input control and expressed as the fold of enrichment relative to the anti-normal rabbit IgG control.

### Measurement of ROS levels

ROS Fluorometric Assay Kit (Elabscience, China) was used to measure the levels of ROS in the spinal cord tissue. Briefly, sheared contused or sham group spinal cord tissue was digested using collagenase/dispase (Sigma, USA), and after erythrocyte lysis and cell counting, 2,7-dichlorofluorescin diacetate (DCFH-DA) reagent was added and incubated for 30 min at 37 °C in the dark, and after washing three times with medium, the cells were resuspended in PBS. Fluorescence was detected by an enzyme marker (Thermo Fisher Scientific, USA) at an excitation wavelength of 500 nm and emission wavelength of 525 nm.

### Phalloidin staining

To visualize cytoskeletal actin, we stained SCMECs using phalloidin [32]. Briefly, the monolayer endothelial cell model was washed with PBS and fixed for 10 min at room temperature using 4% formaldehyde solution. and permeabilized with 0.5% Triton X-100 solution for 5 min. The cells were incubated with phalloidin (100 nM, Solarbio, China) for 30 min at room temperature in the dark. Cells were then re-stained with 4',6-diamidino-2-phenylindole (DAPI) for nuclear labeling. Fluorescence microscopy was used for the analysis (Zeiss Apotome 2, JPN).

### Immunofluorescence

After being treated with 0.5% TritonX-100 in PBS for 30 min at room temperature, tissue or cell samples were sealed with 5% BSA in PBS for 1 h. The primary antibody should then be incubated at 4 °C for a second day. The slices were incubated with the secondary antibody for 1.5 h after being washed with PBS. The sections were then mounted using FluoroshieldTM (GeneTex Inc.), which contains DAPI. Fluorescence microscopy was used for the analysis (Zeiss Apotome 2, JPN). Using ImageJ software, the analyses' findings were quantified.

We utilized the following primary antibodies, secondary antibodies and dilutions: goat anti-CD31 Alexa Fluor 488-conjugated antibody (1:200, R&D, USA), rabbit anti-UTX (1:100, MilliporeSigma, USA), rabbit anti-Claudin5 (1:100, Abcam, USA), rabbit anti-Occludin (1:100, CST, USA), rabbit anti-Zona occludens 1(ZO-1) (1:200, Invitrogen, USA), rat anti-F4/80(1:200, Abcam, USA), rabbit MLCK (1:200, Abcam, USA), rabbit anti-p-MLC (1:50, CST, USA), and anti-goat Alexa Fluor 488 or anti-rabbit / anti-rat Alexa Fluor 594 (1: 400, Abcam, USA).

### Western blotting

Total protein was extracted by lysis in radio immunoprecipitation assay (RIPA, Sigma, USA) lysis buffer. After measuring the protein concentration, transferred to polyvinylidene fluoride membranes (PVDF, Millipore, Billerica, MA) following resolution with SDS-polyacrylamide gel. Finally, data were verified using a ChemiDoc XRS Plus luminescent image analyzer (Bio-Rad, USA) and enhanced chemiluminescence reagents (MIKX, China). We utilized the following primary antibodies and dilutions: rabbit anti-Claudin5 (1:1000, Abcam, USA), rabbit anti-Occludin (1:1000, CST, USA), rabbit anti-ZO-1 (1:1000, Invitrogen, USA), rabbit anti-MLCK (1:1000, Proteintech, USA), rabbit anti-MLC (1:1000, Abcam, USA) and rabbit anti-p-MLC (1:1000, CST, USA). Blots were incubated with peroxidase-conjugated goat anti-rabbit IgG (1:5000, CST, USA) after being rinsed with Tris-buffered saline with Tween (TBST). A rabbit anti-β-actin antibody (1:5000, CST, USA) was used to check for equal protein loading.

### Quantitative real-time PCR (qRT-PCR) analysis

Total RNA was extracted from tissues and cells using Trizol reagent (Invitrogen, USA) and then reverse transcribed using the GoScript™ Reverse Transcription System reverse transcription kit (Promega, USA). Real-time fluorescence qPCR was performed using the GoScript™ qPCR Master Mix kit (Promega, USA) according to the instructions. GAPDH was used as an internal reference and the corresponding gene expression was calculated using the 2-ΔΔCT method. Table 1 shows the primer sequences used in qRT-PCR.

**Table 1:** All primer sequences used for qRT-PCR

Claudin5	Forward primer	GCAAGGTGTATGAATCTGTGCT
	Reverse primer	GTCAAGGTAACAAAGAGTGCCA
Occludin	Forward primer	TGGGCAGTCGGGTTGACT
	Reverse primer	GGGCATCATGGTGTTCATTG
ZO-1	Forward primer	GCCGCTAAGAGCACAGCAA
	Reverse primer	TCCCCACTCTGAAAATGAGGA
MLCK	Forward primer	CCATATCCGAGAAATGCTGGG
	Reverse primer	TGGGATTCCAGGTATGTATCACC
Akap12	Forward primer	CTGTCTGCCGTCAATGGTGTA
	Reverse primer	TGAAGCAGGGATCTGTTCGAT
Bmp6	Forward primer	AGAAGCGGGAGATGCAAAAGG
	Reverse primer	GACAGGGCGTTGTAGAGATCC

### Statistical analysis

All experimental data were processed using the statistical analysis software SPSS 22.0 and results are presented as mean ± standard deviation (*x* ± *s*). The Pearson correlation coefficient was used for the correlation analysis, with r values ranging from − 1 to 1. The correlation (positive/negative correlation) is stronger the closer the absolute value is to 1. An unpaired t-test was used to compare two groups, and a paired t-test was used to compare pre- and post-treatment values. To analyze differences between three or more groups or between groups over time, one-way or two-way ANOVA with Tukey's post hoc test was utilized. Statistical significance was defined as a value of *p* < 0.05.

## Result

### The increase in BSCB permeability parallels elevated UTX expression post-SCI

**Fig. 1:**
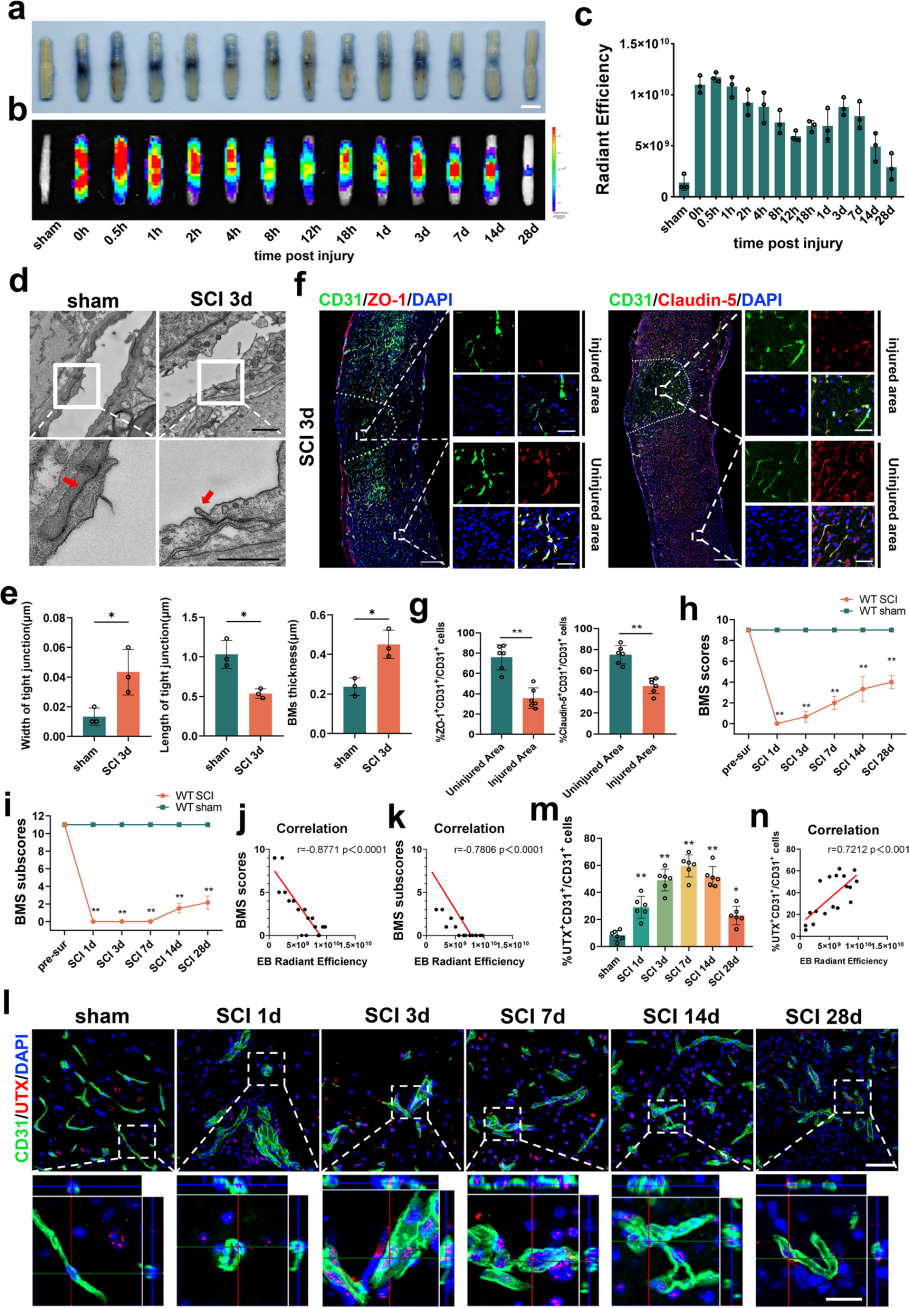
The increase in BSCB permeability parallels elevated UTX expression post-SCI. **a**, **b** Representative digital pictures and NIRF images of spinal cord samples from EB leakage tests taken before and after SCI in WT mice. Scale bar, 5 mm. **c** Quantitative analysis of the radiant efficiency of EB in **b**. *n* = 3 per group. **d** Representative TEM images of WT mice SCI center ECs TJs in the sham and SCI 3d group. Red arrows point to TJs. Scale bar, 1 μm. **e** Quantitative analysis of TJs width, length and BMs thickness in **d**. *n* = 3 per group. **f** Representative immunofluorescence images of ZO-1 or Claudin-5 (red) and CD31 (green), and DAPI (blue) staining of the spinal cord injured and uninjured regions in WT mice with SCI 3d. Scale bars, 500 μm and 50 μm. **g** Quantitative analysis of CD31^+^ZO-1^+^ and CD31^+^Claudin-5^+^ cells as a percentage of CD31^+^ cells in **f**, *n* = 6 per group. **h**–**i** Distribution of the BMS scores and BMS sub-scores in sham and after SCI throughout the 28-day period. n = 12 per group. **j**, **k** Pearson correlation analysis of radiant efficiency of EB leakage in **c** at the corresponding time point with BMS scores and BMS sub-scores in **h**, **i**. *r* = − 0.8771, *P* < 0.0001 and *r* = − 0.7806, *P* < 0.0001. **l** Representative immunofluorescence images of UTX (red), CD31 (green) and DAPI (blue) staining of SCI centers at different time points before SCI and after SCI. Scale bars, 50 μm and 20 μm. **m** Quantitative analysis of CD31^+^UTX^+^ cells as a percentage of CD31^+^ cells in **l**, *n* = 6 per group compared with the corresponding sham group. **n** Pearson's correlation analysis of the fluorescence intensity of EB leakage in the corresponding time point **c** plot with the percentage of CD31^+^UTX^+^ cells to CD31^+^ cells in **l**, *r* = 0.7212, *P* < 0.001. Data are represented as mean ± SEM. *P < 0.05, **P < 0.01

To elucidate the relationship between altered BSCB permeability and post-SCI neurological function, we investigated the spatiotemporal pattern of BSCB permeability alterations in a wild-type mouse SCI model using the Evans Blue leakage assay, at multiple consecutive time points following SCI (sham, 0 h, 0.5 h, 1 h, 2 h, 4 h, 8 h, 12 h, 18 h, 1d, 3d, 7d, 14d, 28d). Our observations revealed a biphasic increase in BSCB permeability following SCI, with the first peak of abnormal leakage occurring within 1 h post-injury and the second peak appearing on day 3 post-injury (Fig. 1a–c). Assessments of EB leakage area and fluorescence intensity of leaked EB were consistent with the above results (Additional file 1: Fig. S1a–c). Drawing from the data aforementioned, we chose the SCI 3d specimens with the most substantial permeability change and employed TEM to investigate the TJ structure between ECs. Our results demonstrated that the TJ gap in the SCI group was significantly wider and shorter than that of the sham group. Moreover, the BMs play a pivotal role in sustaining BSCB function[33, 34]. Our results found that the BMs of the vascular endothelium was notably thicker in the SCI 3d group than in the sham group, indicating a disruption in the BSCB structure (Fig. 1d, e). Additionally, the immunofluorescence results indicate a significant reduction in the number of ZO-1^+^CD31^+^ and Claudin-5^+^CD31^+^ cells in the SCI 3d group injury area, as compared to the non-injured area (see Fig. 1f, g). The outcomes from Western blotting and qRT-PCR further confirm the diminished expression levels of Claudin-5, Occludin, and ZO-1 were downregulated in the center of the SCI (see Additional file 1: Fig. S1d–f).

Post-SCI, our hindlimb motor functionality assessments highlighted significantly depressed BMS scores and sub-scores in comparison to the sham group (Fig. 1h. i). A Pearson correlation analysis further unveiled a strong negative association between the hind limb motor function scores and the trajectory of permeability changes (Fig. 1j, k). We also found an upsurge in UTX expression within vascular ECs post-SCI, culminating on day 7, echoing findings from our prior study (Fig. 1l, m) [25]. Importantly, the trend of UTX expression after SCI exhibited a significant positive correlation with the trend of permeability alteration (Fig. 1n). These findings indicate a close association between BSCB permeability after SCI and UTX expression.

In our in vitro experiments, we isolated and cultured SCMECs from WT mice and subjected them to OGD treatment (Additional file 1: Fig. S2a–c). Our findings indicate that SCMECs showed significantly higher penetration in the OGD group (Additional file 1: Fig. S2d–f), and the TJs were disrupted (Additional file 1: Fig. S2g–j).

### Endothelial-specific UTX knockout enhances BSCB integrity and diminishes both macrophage infiltration and ROS levels post-SCI

**Fig. 2:**
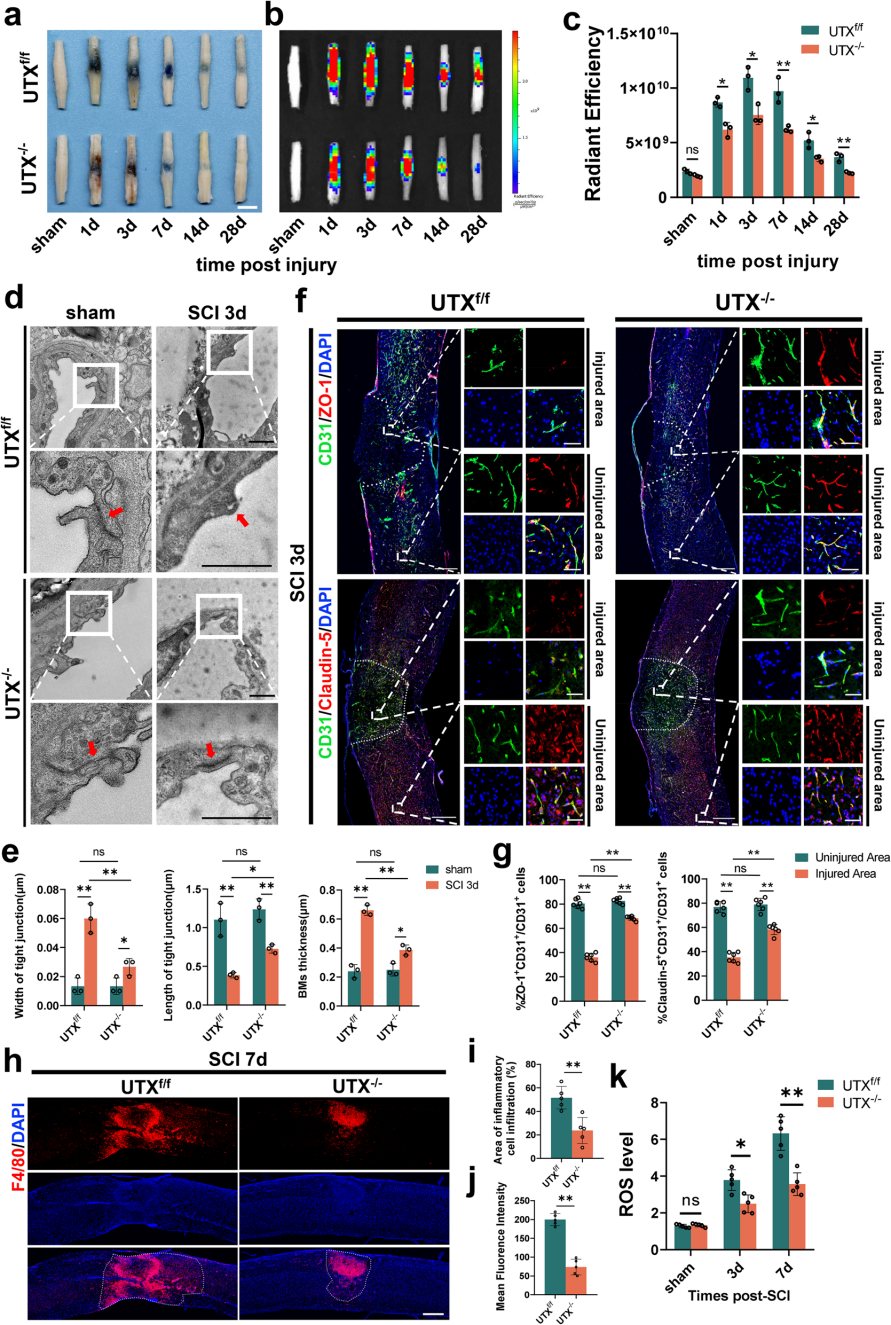
Endothelial-specific UTX knockout enhances BSCB integrity and diminishes both macrophage infiltration and ROS levels post-SCI**.** **a**, **b** Representative digital pictures and NIRF images of spinal cord samples from EB leakage tests taken before and after SCI at different time points in UTX^f/f^ and UTX^−/−^ mice. Scale bar, 5 mm. **c** Quantitative analysis of the radiant efficiency of EB in **b**. *n* = 3 per group. **d** Representative TEM images of UTX^f/f^ and UTX^−/−^ mice SCI center ECs TJs in the sham and SCI 3d group. Red arrows point to TJs. Scale bar, 1 μm. **e** Quantitative analysis of TJs width, length and BMs thickness in **d**. *n* = 3 per group. **f** Representative immunofluorescence images of ZO-1 or Claudin-5 (red) and CD31 (green), and DAPI (blue) staining of the spinal cord injured and uninjured regions in UTX^f/f^ and UTX^−/−^ mice with SCI 3d. Scale bars, 500 μm and 50 μm. **g** Quantitative analysis of CD31^+^ZO-1^+^ and CD31^+^Claudin-5^+^ cells as a percentage of CD31^+^ cells in **f**, *n* = 6 per group. **h** Representative immunofluorescence images of UTX^f/f^ and UTX^−/−^ mouse SCI 7d spinal cord specimens stained with F4/80 and DAPI (blue). Scale bar, 500 μm. **i**, **j** Quantitative evaluation of F4/80 cells infiltration area and mean fluorescence intensity in **h**. *n* = 5 per group. **k** ROS levels in the epicenter of the SCI area. Data are represented as mean ± SEM. ns *P* > 0.05, *P < 0.05, **P < 0.01

To better understand whether UTX is involved in regulating the altered BSCB permeability after SCI. The mice with endothelial cell-specific deficiency of UTX were used in our study (Additional file 1: Fig. S3a-c). We undertook EB leakage tests, demonstrating markedly reduced EB leakage in UTX^−/−^ mice relative to the UTX^f/f^ mice at the same time points (Fig. 2a–c). Fluorescence imaging further corroborated these observations (Additional file 1: Fig. S3d–f). The results of transmission electron microscopy revealed no significant differences between the UTX^−/−^ sham group and the UTX^f/f^ sham group. However, at 3 days after SCI, UTX^−/−^ mice exhibited attenuated disruption of TJs. Additionally, UTX^−/−^ mice exhibited an increase in the length of TJs and a decrease in the thickness of the vascular endothelial basement membrane (Fig. 2d, e). Further analysis using immunofluorescence indicated that the injured area of UTX^−/−^ mice had a significantly higher number of ZO-1^+^CD31^+^ and Claudin-5^+^CD31^+^ cells than the UTX^f/f^ group (Fig. 2f, g). In addition, our results found that there was no statistically significant difference in microvessel density and microvessel diameter between the UTX^−/−^ and UTX^f/f^ groups before injury. After SCI, the microvessel density was significantly higher in the UTX^−/−^ group than in the UTX^f/f^ group, but there was no significant difference in microvessel diameter (Additional file 1: Fig. S3g, h). Moreover, Western blotting and qRT-PCR results also showed that the expression levels of Claudin-5, Occludin, and ZO-1 were significantly higher in the injured epicenter of UTX^−/−^ mice at 3 days post-SCI (Additional file 1: Fig. S3i, m).

Oxidative stress and inflammatory cell infiltration are important drivers of the inflammatory microenvironment after SCI [35–37]. We selected spinal cord specimens taken 7 days after SCI for immunofluorescent staining of macrophages, as this time point coincides with the peak of macrophage aggregation [38]. The ROS levels were also measured using the DCFH-DA assay. The findings demonstrate a notable decline in macrophage infiltration within the UTX^−/−^ group (Fig. 2h–j), coupled with a significant decrease in ROS levels (Fig. 2k). Therefore, the gathered data underscore that endothelial-specific UTX knockout mitigates BSCB permeability issues, while concurrently curtailing inflammatory infiltration and ROS concentrations post-SCI in mice.

### Endothelial UTX knockout diminished BSCB permeability post-OGD exposure

**Fig. 3:**
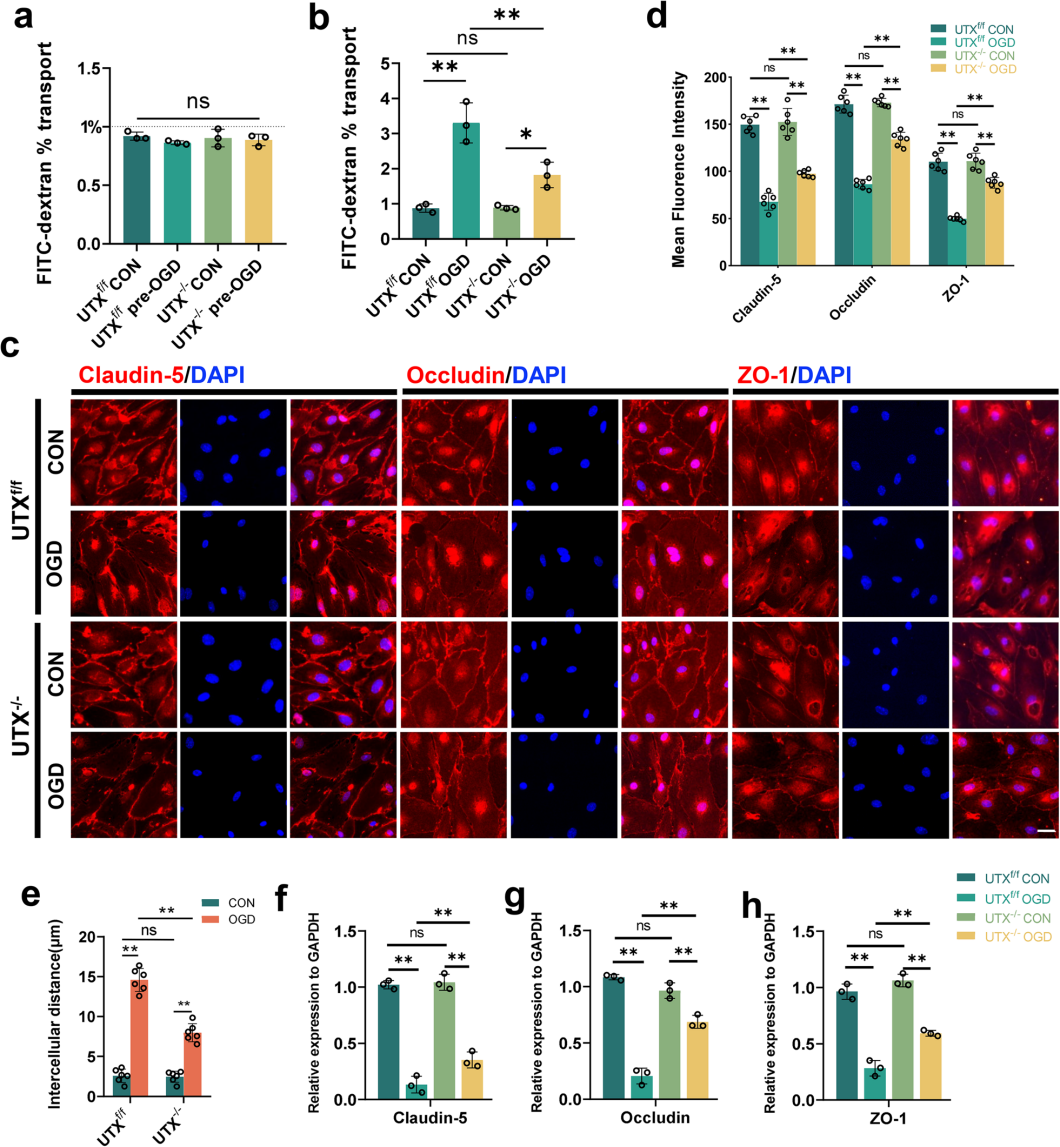
Endothelial UTX knockout diminished BSCB permeability post-OGD exposure. **a**, **b** FITC-dextran transports assay the permeability of UTX^f/f^ and UTX^−/−^ SCMECs at pre- and post-OGD. *n* = 3 per group. **c** Representative immunofluorescence images of TJs-related protein (Claudin-5, Occludin, and ZO-1) in the UTX^f/f^ and UTX^−/−^ SCMECs when exposed to OGD. Scale bar, 20 μm. **d** Quantitative evaluation of the fluorescence intensity of Claudin-5, Occludin, and ZO-1 in **c**. *n* = 6 per group. **e** Quantitative evaluation of intercellular distance in **c**. *n* = 6 per group. **f**–**h** qRT-PCR verification of the mRNA levels of Claudin-5, Occludin, and ZO-1 in the UTX^f/f^ and UTX^−/−^ SCMECs when exposed to OGD. n = 3 per group. Data are represented as mean ± SEM. ns *P* > 0.05, *P < 0.05, **P < 0.01

To investigate the role of UTX in regulating altered ECs permeability and oxidative stress levels after OGD treatment in vitro, SCMECs were isolated and propagated from UTX^f/f^ and UTX^−/−^ mice. Subsequently, we established transwell monolayer endothelial models and executed permeability tests using FITC-dextran introduced into the superior chamber. Pre-OGD, the cell permeability of all four groups was lower than 1%, and there was no significant difference observed (Fig. 3a). Post-OGD exposure, a pronounced surge in FITC-dextran permeability was evident in both the UTX^f/f^ OGD and UTX^−/−^ OGD groups. Notably, the permeability levels in the UTX^−/−^ OGD set were substantially attenuated relative to the UTX^f/f^ OGD ensemble, hinting at UTX's pivotal role in endothelial cell-to-cell adhesion (Fig. 3b). The immunofluorescent assessments for Claudin-5, Occludin, and ZO-1 showcased the UTX^−/−^ OGD group boasting a markedly intensified TJs fluorescence, while exhibiting reduced cellular gaps compared to the UTX^f/f^ OGD group (Fig. 3c–e). qRT-PCR evaluations resonated with these observations (Fig. 3f–h). These findings suggest that the ablation of UTX mitigates the disruption of TJs, and endothelial intercellular permeability following OGD.

### Endothelial-specific UTX knockout enhances neurological recovery in mice after SCI

**Fig. 4:**
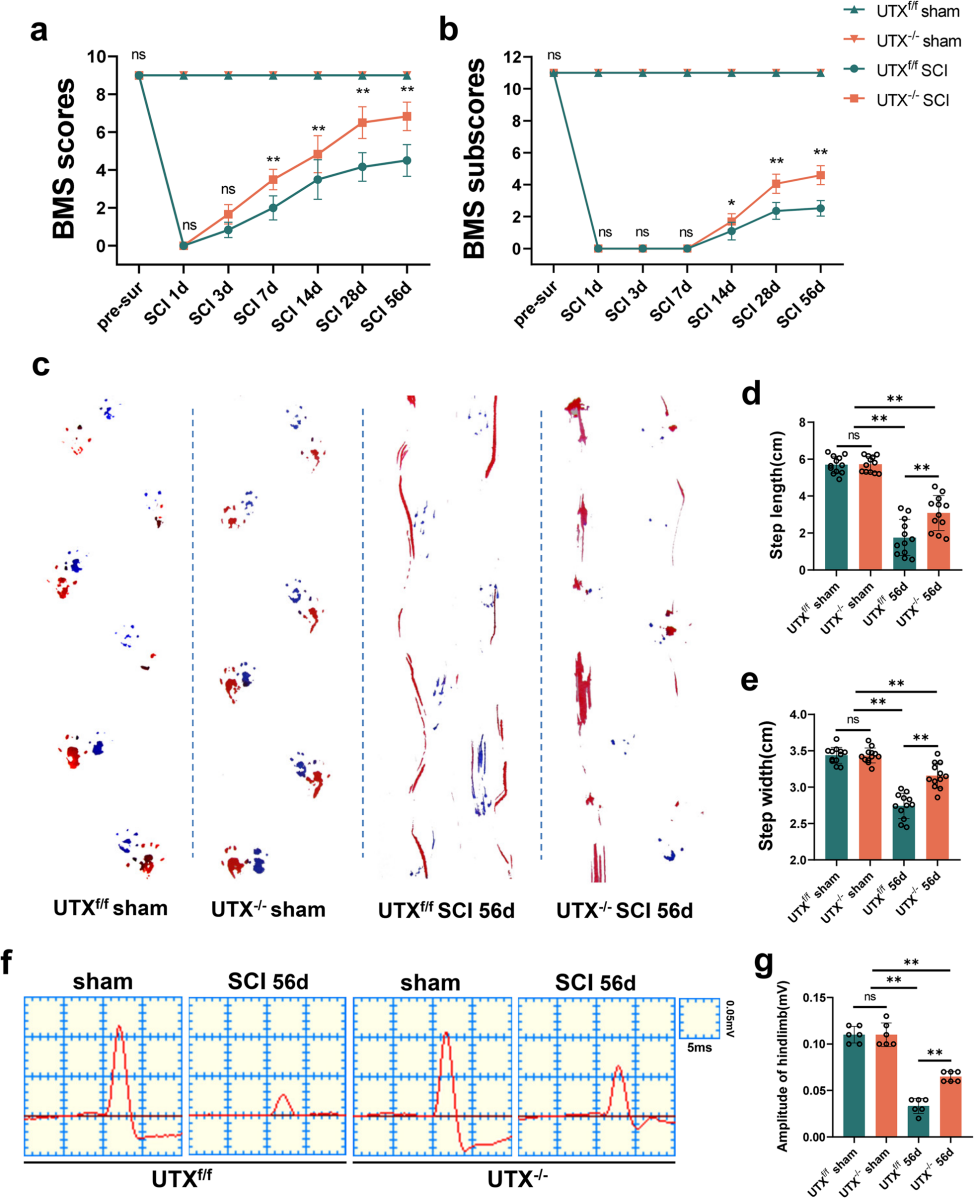
Endothelial-specific UTX knockout enhances neurological recovery in mice after SCI. **a**, **b** Distribution of the BMS scores and BMS sub-scores in UTX^f/f^ and UTX^−/−^ groups at pre-surgery and after SCI throughout the 56-day period. *n* = 12 per group. **c** Typical digital photographs of the gait analysis of the mice in UTX^f/f^ and UTX^−/−^ groups at sham and on days 56 pre-SCI. Forelimbs: blue dyes, hindlimbs: red dyes. **d**, **e** Quantitative evaluation of the width and length of gait per a step in **c**. *n* = 12 per group. **f** Representative images of motor-evoked potential (MEP) in sham, in UTX^f/f^ and UTX^−/−^ groups at 56-day post-SCI. **g** Quantitative evaluation of the amplitude of MEPs in **f**. *n* = 6 per group. Data are represented as mean ± SEM. ns *P* > 0.05, *P < 0.05, **P < 0.01

We further assessed the impact of endothelial-specific UTX knockout on post-SCI neurological recuperation. Initially, motor function in the hind limbs of UTX^f/f^ and UTX^−/−^ mice was measured at various time points after SCI using BMS scores. Results showed no alteration in BMS scores and sub-scores in the UTX^f/f^ sham and UTX^−/−^ sham groups, indicating that UTX ablation does not impact normal hindlimb motor function in mice. Conversely, UTX^−/−^ mice demonstrated significantly higher BMS scores and sub-scores at 7, 14, 28, and 56 days post-SCI compared to UTX^f/f^ mice, suggesting that UTX knockout in ECs promotes hindlimb motor function recovery after SCI (Fig. 4a, b). Additionally, Catwalk analysis revealed that UTX^−/−^ mice exhibited a significantly better gait than UTX^f/f^ mice at 56 days post-SCI (Fig. 4c). Quantitative analysis indicated that gait width and length were significantly greater in the UTX^−/−^ group (Fig. 4d, e). Furthermore, at 56 days post-SCI, UTX^−/−^ mice displayed a higher amplitude of motor-evoked potentials (MEPs) (Fig. 4f, g). Collectively, this data emphasizes that endothelial-targeted UTX ablation confers notable neurological advantages in a SCI mouse model.

### UTX plays a pivotal role in regulating MLCK expression

**Fig. 5:**
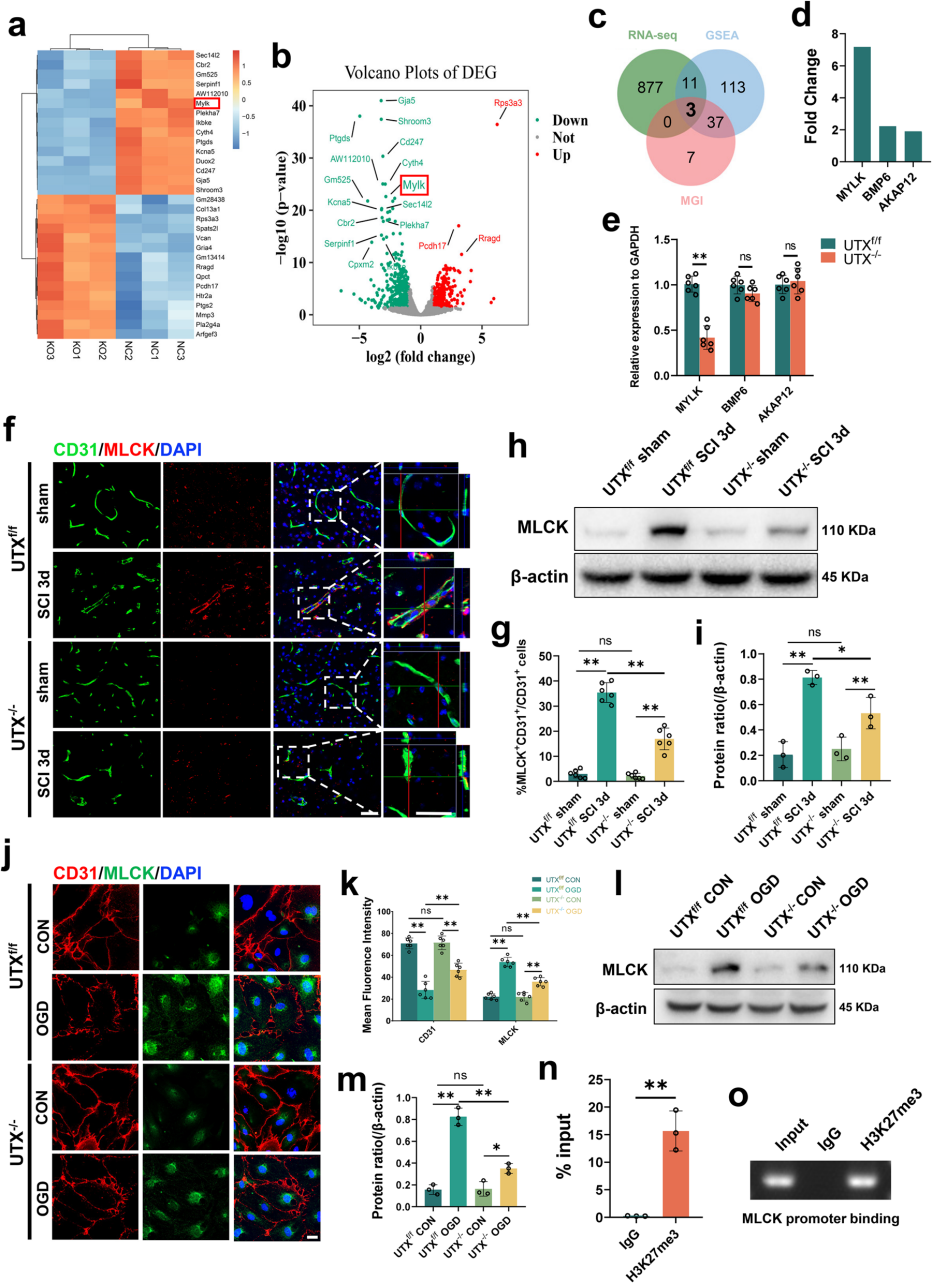
UTX plays a pivotal role in regulating MLCK expression. **a**, **b** UTX^f/f^ SCMECs Vs UTX^−/−^ SCMECs were significantly different (*p* ≤ 0.05, logFC ≥ 1 or ≤ − 1) from heatmap and volcano map of RNA expression profiles. **c** Venn diagram of the intersection of differential genes screened by RNA-seq and genes associated with vascular permeability retrieved from GSEA and MGI databases. **d** Differential ploidy of the genes screened in **c**. **e** qRT-PCR verification of the mRNA levels of MYLK (MLCK), BMP6, and AKAP12 in the UTX^f/f^ and UTX^−/−^ mice at 3 days post-SCI. n = 3 per group. **f** Representative immunofluorescence images of MLCK (red) and CD31 (green), DAPI (blue) staining of injured epicenter of spinal cord at SCI 3d. Scale bars, 50 μm. **g** Quantitative analysis of CD31^+^MLCK^+^ cells as a percentage of CD31^+^ cells in **f**, n = 6 per group. **h** Western blotting analysis of MLCK protein expression levels in UTX^f/f^ and UTX^−/−^ mice at sham and SCI 3d. **i** Quantitative analysis of the expression levels of MLCK in **h**. *n* = 3 per group. **j** Representative immunofluorescence images of MLCK (red) and CD31 (green), DAPI (blue) staining in UTX^f/f^ and UTX^−/−^ SCMECs after OGD. Scale bars, 20 μm. **k** Quantitative analysis of the fluorescence intensity of CD31 and MLCK in **j**, *n* = 6 per group. **l** Western Blotting analysis of MLCK protein expression levels in UTX^f/f^ and UTX^−/−^ SCMECs after OGD. **m** Quantitative analysis of the expression levels of MLCK in **l**. *n* = 3 per group. **n** ChIP-qPCR to detect the binding rate of histone H3K27me3 to MLCK initiation sequence. *n* = 3 per group. **o** Gel electrophoresis diagram of ChIP-qPCR products. Data are represented as mean ± SEM. ns *P* > 0.05, *P < 0.05, **P < 0.01

To probe the mechanistic foundation behind the modified BSCB permeability post-conditional UTX deletion in ECs, we extracted SCMECs from both UTX^f/f^ mice and UTX^−/−^ mice. Analysis of bulk RNA sequencing data revealed differential expression of 891 genes between SCMECs from UTX^f/f^ and UTX^−/−^ mice, of which 439 were upregulated and 452 were downregulated (with p ≤ 0.05 and logFC ≥ 1 or ≤ − 1) (Fig. 5a, b). We then searched for genes associated with "vascular permeability" in the GSEA database (http://www.gsea-msigdb.org/gsea/index.jsp) and MGI databases (https://www.Informatics.jax.org) and identified three target genes: MYLK(MLCK), BMP6, and AKAP12 (Fig. 5c, d). Further qRT-PCR tests revealed that MLCK was considerably differentially expressed in the injury centers of UTX^f/f^ and UTX^−/−^ mice 3 days after SCI, whereas BMP6 and AKAP12 were not significantly different (Fig. 5e). Subsequently, we confirmed MLCK expression both in vivo and in vitro. The results of the immunofluorescence analysis revealed a very significant increase in the number of CD31^+^MLCK^+^ cells in UTX^f/f^ mice 3 days SCI when compared to their respective sham groups, as demonstrated in Fig. 5f, g. In contrast, the changes observed in UTX^−/−^ mice were less significant compared to those observed in UTX^f/f^ mice. The Western blotting results showed a similar trend (Fig. 5h, i), which was also confirmed in vitro (Fig. 5j–m). To explore the underlying mechanism, we performed ChIP-qPCR and found that H3K27me3 was significantly enriched in the promoter region of MCLK in UTX^f/f^ SCMECs after OGD (Fig. 5n, o), indicating a possible physical binding between H3K27me3 and the MLCK promoter region. Taken together, these findings suggest that UTX regulates MLCK expression through an epistatic regulatory complex involving histone H3K27me3 and MLCK.

### MLCK is integral to EC permeability modulation following in vitro OGD treatment

**Fig. 6:**
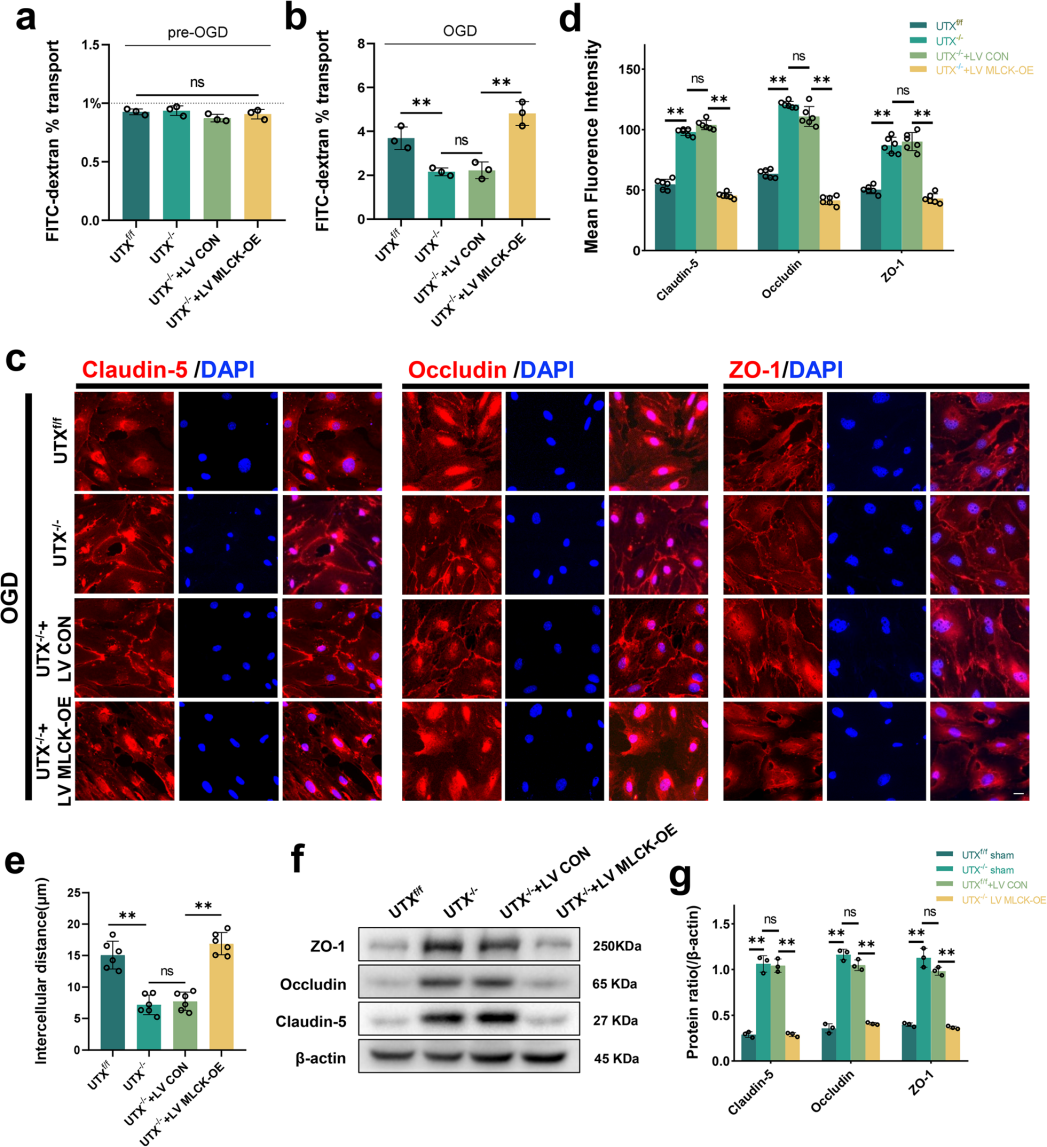
MLCK is integral to EC permeability modulation following in vitro OGD treatment. **a**, **b** FITC-dextran transports assay the permeability of UTX^f/f^, UTX^−/−^, UTX^−/−^ + LV CON, and UTX^−/−^ + LV MLCK-OE SCMECs at pre- and post-OGD. *n* = 3 per group. **c** Representative immunofluorescence images of TJs-related protein (Claudin-5, Occludin, and ZO-1) in the UTX^f/f^, UTX^−/−^, UTX^−/−^ + LV CON, and UTX^−/−^ + LV MLCK-OE SCMECs when exposed to OGD. Scale bar, 20 μm. **d** Quantitative evaluation of the fluorescence intensity of Claudin-5, Occludin and ZO-1 in **c**. *n* = 6 per group. **e** Quantitative evaluation of intercellular distance in **c**. n = 6 per group. **f** Western blotting analysis of the TJs-related protein levels including ZO-1, Occludin, and Claudin-5 in UTX^f/f^, UTX^−/−^, UTX^−/−^ + LV CON, and UTX^−/−^ + LV MLCK-OE SCMECs after OGD. **g** Quantitative analysis of the expression levels of ZO-1, Occludin, and Claudin-5 in **f**. *n* = 3 per group. Data are represented as mean ± SEM. ns *P* > 0.05, **P < 0.01

To confirm the role of MLCK-mediated modulation of vascular permeability after OGD treatment, we transfected SCMECs from UTX^−/−^ mice with MLCK overexpression lentivirus (Additional file 1: Fig. S4a). Before OGD treatment, the cell permeability in all groups, including UTX^f/f^ group, UTX^−/−^ group, UTX^−/−^ + LV CON group, and UTX^−/−^ + LV MLCK-OE group, was less than 1% with no discernible difference. Post-OGD treatment, the FITC-dextran permeability significantly increased in the UTX^f/f^ group. Contrastingly, the UTX^−/−^ and UTX^−/−^ + LV CON groups presented reduced permeability. This decrement was negated in the UTX^−/−^ + LV MLCK-OE ensemble (Fig. 6a, b). Immunofluorescence assays highlighted that the UTX^−/−^ and UTX^−/−^ + LV CON groups had markedly amplified fluorescence intensities of Claudin-5, Occludin, and ZO-1 compared to the UTX^f/f^ cohort. Yet, this trend was counteracted in the UTX^−/−^ + LV MLCK-OE group (Fig. 6c–e). According to Western blotting data, TJs protein expression was significantly higher in the UTX^−/−^ and UTX^−/−^ + LV CON groups compared to the UTX^f/f^ group, while it was decreased in the UTX^−/−^ + LV MLCK-OE group (Fig. 6f, g). The gathered evidence underscores MLCK's pivotal role in governing intercellular permeability within SCMECs post-OGD treatment.

To visualize cytoskeletal actin, SCMECs were stained using phalloidin. The results showed that in the UTX^f/f^ and UTX^−/−^ groups, which were not treated with OGD, a uniform distribution of actin was visible in the cells. However, post-OGD, the UTX^f/f^ set showed conspicuous actin stress fiber genesis, a feature absent in the UTX^−/−^ group. In addition, UTX^f/f^ + ML7 (an MLCK inhibitor) gave the same results as the UTX^−/−^ group (Additional file 1: Fig. S5a, b). The data intimate that UTX may orchestrate actin polymerization via the MLCK/p-MLC pathway, precipitating BSCB disturbances.

### MLCK overexpression mitigated reduced BSCB permeability in UTX^−/−^ mice

**Fig. 7:**
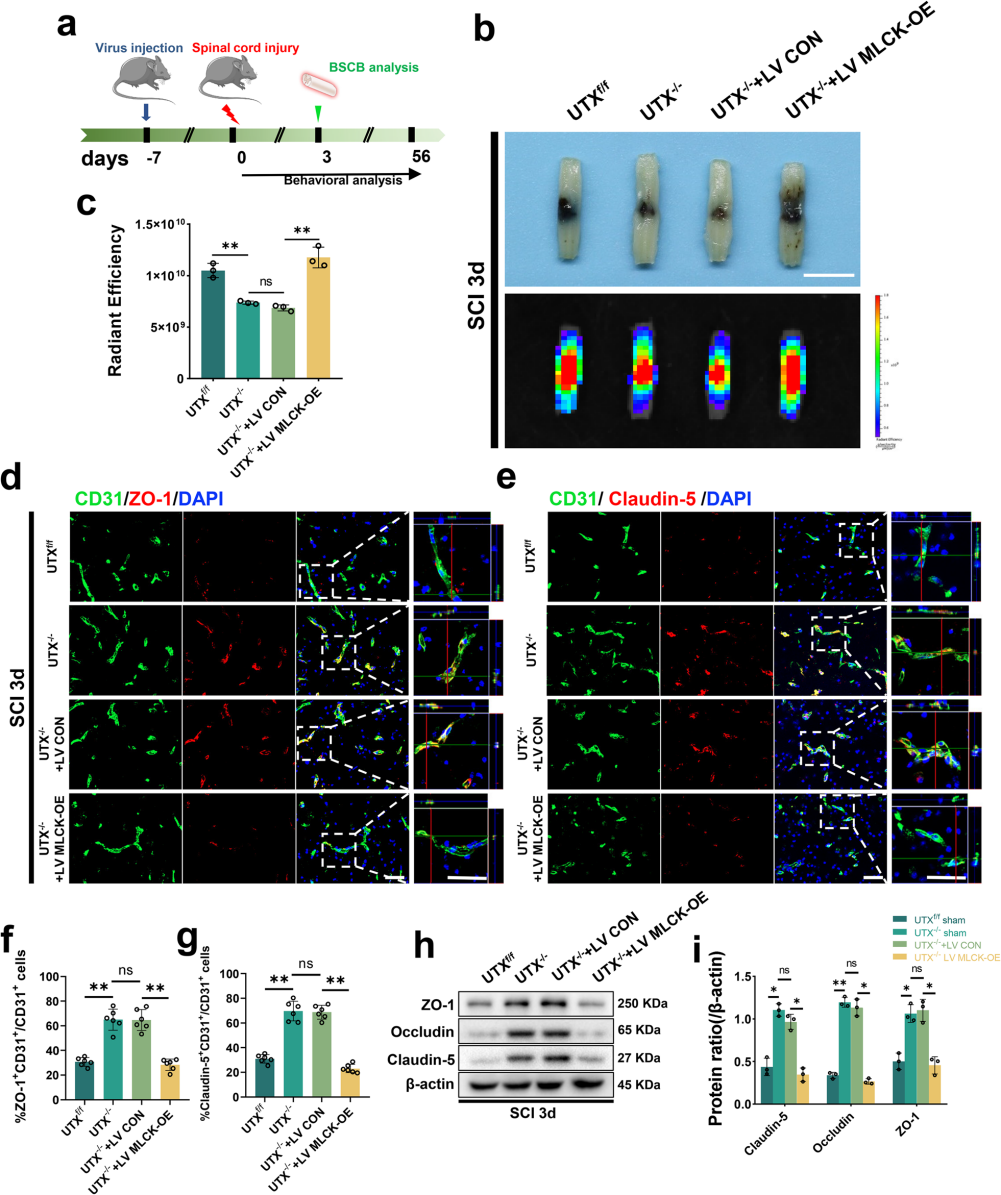
MLCK overexpression mitigated reduced BSCB permeability in UTX^−/−^ mice. **a** Experimental scheme. Lentivirus was injected into the spinal cord 7 days prior to SCI, and SCI 3d was performed for vascular permeability testing and further testing for neurological function analysis. **b** Representative digital pictures and NIRF images of spinal cord samples from EB leakage tests taken SCI 3d in UTX^f/f^, UTX^−/−^, UTX^−/−^ + LV CON, and UTX^−/−^ + LV MLCK-OE mice. scale bar, 5 mm. **c** Quantitative analysis of the radiant efficiency of EB in **b**. *n* = 3 per group. **d**, **e** Representative immunofluorescence images of ZO-1 or Claudin-5 (red) and CD31 (green), and DAPI (blue) staining of the injured epicenter of spinal cord in UTX^f/f^, UTX^−/−^, UTX^−/−^ + LV CON, and UTX^−/−^ + LV MLCK-OE mice with SCI 3d. Scale bars, 50 μm. **f**, **g** Quantitative analysis of CD31^+^ZO-1^+^ and CD31^+^Claudin-5^+^ cells as a percentage of CD31^+^ cells in **d**, **e**, *n* = 6 per group. **h** Western blotting analysis of the TJs-related protein levels including ZO-1, Occludin, and Claudin-5 in UTX^f/f^, UTX^−/−^, UTX^−/−^ + LV CON, and UTX^−/−^ + LV MLCK-OE mice at SCI 3d. **i** Quantitative analysis of the expression levels of ZO-1, Occludin, and Claudin-5 in **h**. *n* = 3 per group. Data are represented as mean ± SEM. ns P > 0.05, *P < 0.05, **P < 0.01

To confirm the efficacy of MLCK overexpression, we transfected the spinal cord with lentivirus via intrathecal injection and observed a peak in transfection efficiency 10 days after intrathecal injection (Additional file 1: Fig. S4b, c). Based on these findings, we designed an experimental approach that involved lentivirus administration 7 days before SCI, evaluation of BSCB permeability at 3 days post-SCI, and subsequent assessment of hindlimb motor function and neurological status (Fig. 7a). Our initial experiments using EB leakage assays revealed significantly lower leakage in the UTX^−/−^ and UTX^−/−^ + LV CON groups than in the UTX^f/f^ group, but leakage was significantly higher in the UTX^−/−^ + LV MLCK-OE group (Fig. 7b, c, Additional file 1: Fig. S4d–f). Further co-staining experiments with ZO-1, Claudin-5, and CD31 antibodies showed thickening and malformation of the vasculature in the injured epicenter of the spinal cord in all groups, but the number of ZO-1^+^CD31^+^ and Claudin-5^+^CD31^+^ cells was significantly higher in the UTX^−/−^ and UTX^−/−^ + LV CON groups than in the UTXf/f group, and this effect was reversed after MLCK overexpression (Fig. 7d–g). The corroboration from Western blot assays mirrored these observations (Fig. 7h, i), underscoring MLCK's role in modulating vascular permeability post-SCI in EC-specific UTX^−/−^ mouse models.

### Conditional UTX knockout in ECs regulates BSCB permeability following SCI via the MLCK/p-MLC pathway, promoting neurological recovery

In ECs, MLCK phosphorylation of MLC instigates actin contraction, cytoskeletal retraction, cytosolic pullback, and TJs dissociation, typifying a standard route for intercellular barrier disruption [17, 39]. Our study's objective was to discern if UTX deletion in ECs can influence vascular permeability post-SCI via the MLCK/p-MLC route.

**Fig. 8:**
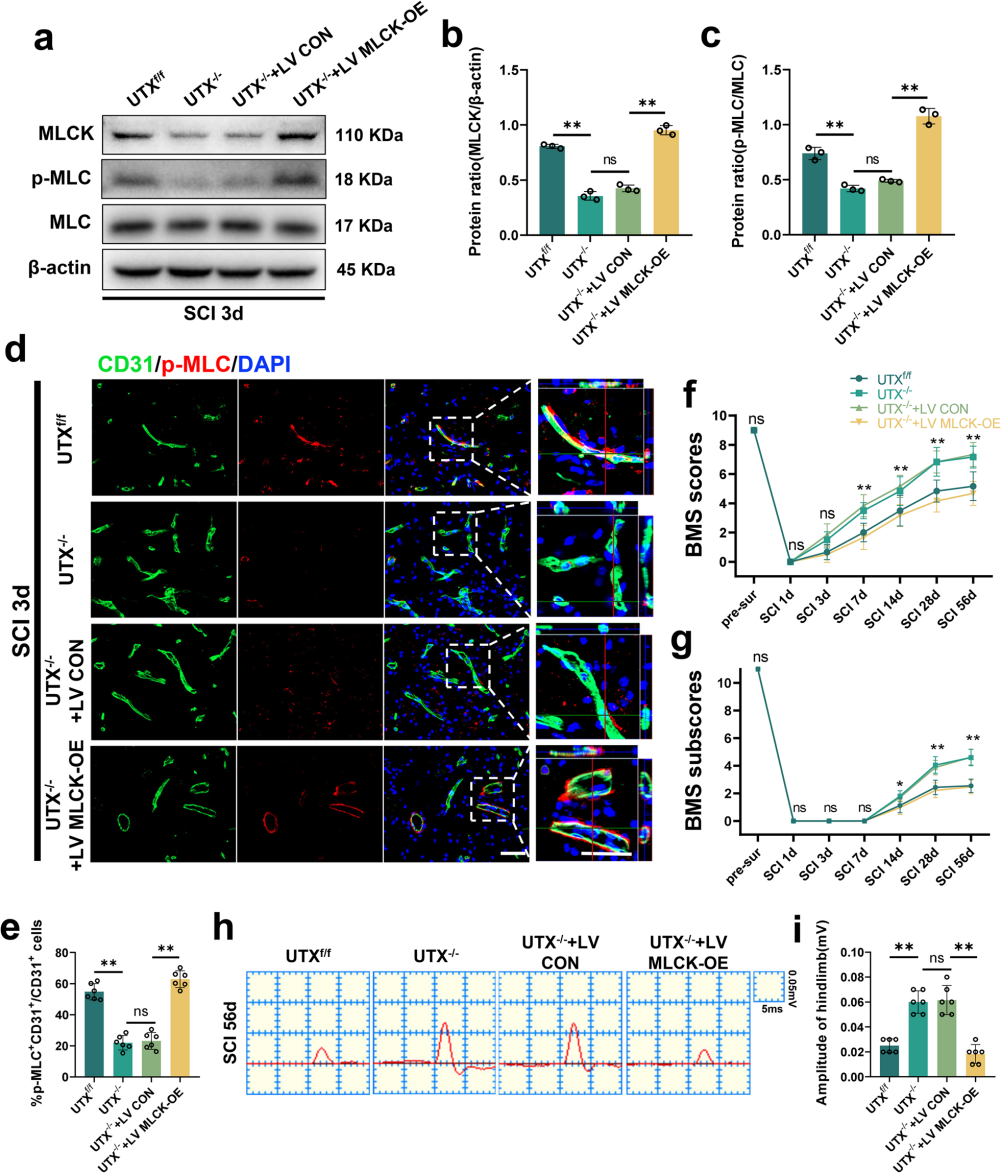
Conditional UTX knockout in ECs regulates BSCB permeability following SCI via the MLCK/p-MLC pathway, promoting neurological recovery. **a** Western blotting analysis of MLCK, p-MLC, MLC in UTX^f/f^, UTX^−/−^, UTX^−/−^ + LV CON, and UTX^−/−^ + LV MLCK-OE mice at SCI 3d. **b**, **c** Quantitative analysis of the expression levels of MLCK and p-MLC in **a**. *n* = 3 per group. **d** Representative immunofluorescence images of p-MLC (red) and CD31 (green), DAPI (blue) staining of injured epicenter of spinal cord in UTX^f/f^, UTX^−/−^, UTX^−/−^ + LV CON, and UTX^−/−^ + LV MLCK-OE mice at SCI 3d. Scale bars, 50 μm. **e** Quantitative analysis of CD31^+^p-MLC^+^ cells as a percentage of CD31^+^ cells in **d**, *n* = 6 per group. **f**, **g** Distribution of the BMS scores and BMS sub-scores in UTX^f/f^, UTX^−/−^, UTX^−/−^ + LV CON, and UTX^−/−^ + LV MLCK-OE mice at pre-surgery and after SCI throughout the 56-day period. *n* = 12 per group. **h** Representative images of motor-evoked potential (MEP) in UTX^f/f^, UTX^−/−^, UTX^−/−^ + LV CON, and UTX^−/−^ + LV MLCK-OE mice at 56-day post-SCI. **i** Quantitative evaluation of the amplitude of MEPs in **h**. *n* = 6 per group. Data are represented as mean ± SEM. ns *P* > 0.05, *P < 0.05, **P < 0.01

The Western blotting results of spinal cord tissues from 3 days post-SCI revealed that the mice in the UTX^f/f^ group had higher levels of the proteins MLCK and p-MLC, while the UTX^−/−^ group had significantly lower levels. However, overexpression of MLCK substantially increased the levels of p-MLC (Fig. 8a–c). The immunofluorescence results demonstrated that MLCK overexpression increased the fluorescence intensity of p-MLC in ECs 3 days post-SCI (Fig. 8d, e), suggesting that the deletion of UTX in ECs promotes BSCB permeability after SCI by blocking the MLCK/p-MLC signaling pathway. Furthermore, mice motor capabilities were evaluated via electrophysiological measures and BMS scoring. Our data suggest that UTX ablation hastens the restoration of hindlimb motility, yet this recuperation is stymied by MLCK upregulation (Fig. 8f, g). Electrophysiologic inspections echoed these sentiments (Fig. 8h, i). Conclusively, the results validate that UTX ablation in ECs markedly augments neurological recuperation in SCI mouse models. In totality, our investigations reveal that conditional UTX ablation in ECs mitigates vascular permeability and catalyzes neurological healing post-SCI by obstructing the MLCK/p-MLC mechanism.

## Discussion

In the present study, our findings reveal that UTX is significantly overexpressed in SCMECs after SCI, and its expression is linked to BSCB dysfunction. Furthermore, in vivo experiments show that specific deletion of UTX in ECs leads to a reduction in BSCB disruption, resulting in less macrophage infiltration and ROS production, as well as improved functional recovery after SCI. Our RNA-seq and ChIP-qPCR analyses revealed that UTX potentially interacts with MLCK, a physical binding partner. The protective effects of endothelial UTX deletion on BSCB after SCI can be attributed to its inhibition of the MLCK/p-MLC pathway. These results suggest that UTX may hold promise as a therapeutic target for treating SCI.

The breakdown of BSCBs is a significant contributor to the challenge and escalating severity of nerve tissue healing after SCI [40, 41]. Vascular ECs injury occurs immediately after SCI, with ruptured blood vessels bleeding at the epicentre shortly after primary injury [42]. The vessel experiences shear forces in the injury's epicenter region, causing structural disruption and raising permeability [5]. Although the neovascularization process also begins 3–4 days after SCI and peaks on 7 [9, 10], these neovascular morphologies are dilated, tortuous, and have unusually high permeability. Therefore, the change in BSCB permeability after SCI is a complicated and multifaceted process. Many questions remain about the laws governing this change, and there are significant discrepancies between the conclusions reached by various researchers [43].

Study indicated that increased BSCB permeability caused by SCI exhibits biphasic waves, with the first peak occurring shortly after the injury and the second peak appearing between 3 and 7 days later [44]. In another study using a rat model, BSCB permeability was found to be significantly improved 14 days after injury, as observed by horseradish peroxidase extravasation [45]. Popovich et al. used the vascular tracer [14C]-alpha-aminoisobutyric acid (AIB) to assess vascular permeability and found elevated AIB leakage in the white matter of the spinal cord 14–28 days after injury [46]. By using EB leakage experiments, Tao Jiang et al. discovered that BSCB permeability substantially increased at 8 h after SCI, peaked at 1 day, and was nearly back to normal at 14 days [47].

Here, we provide a comprehensive understanding of BSCB permeability changes following SCI. We utilized an in vivo imaging system to quantify the extent of extravasated Evans Blue (EB), which allowed for a more precise and visually appealing observation of BSCB permeability changes compared to previous studies that used trichloroacetic acid or formamide solutions for lysis [17]. Our findings revealed two obvious peaks in BSCB permeability alterations following SCI, with the first occurring at 1 h post-injury and the second peaking at three days post-injury, which was consistent with prior findings [44]. Notably, we observed that the initial EB leakage was not confined to the epicenter of the injury, suggesting that the injury-induced vascular rupture led to direct leakage of EB at the epicenter, and structural disruption of the vasculature due to shear forces that increased permeability in the surrounding areas. The subsequent decrease in EB infiltration over the next 12 h may be attributed to the self-repair of damaged vessels, while the increase in EB leakage after 12 h coincided with the trend of post-SCI angiogenesis [9, 10], indicating the development of dilated and convoluted neovascularization with abnormally high permeability.

We also found a significant negative association between hind limb motor function scores and BSCB permeability changes, and a significant positive correlation between BSCB permeability and UTX expression, suggesting that UTX may play a crucial role in influencing BSCB permeability after SCI. Overall, our study provides a more continuous and comprehensive understanding of BSCB permeability changes following SCI and highlights the potential role of UTX in modulating BSCB permeability.

Epigenetic regulations are crucial to axonal regeneration, glial activation, neural stem cell differentiation, and the regeneration and stabilization of spinal microvasculature. Therefore, understanding the changes in epigenetic factors is essential to decoding the mechanisms of SCI recovery [48–50]. For instance, studies have shown that Jumonji domain-containing protein-3 (Jmjd3) mediates BSCB destruction after SCI by regulating the expression of MMP-3 and MMP-9 [16], and Jmjd3 can also be inhibited by gallic acid, thereby ameliorating BSCB destruction after SCI [15]. Additionally, another study found that NOGOB receptor (NGBR) deficiency can enhance cerebrovascular permeability by reducing histone acetylation-mediated cavernous malformation 1/2 (CCM1/2) expression [17]. In our previous research, we investigated the function of UTX in SCI and discovered that UTX expression is increased in neural stem cells, and vascular ECs after SCI. We found that down-regulating or deletion of UTX can enhance neurological recovery after SCI in mice, indicating that UTX plays a significant role in SCI [25, 51, 52]. However, the influence of UTX on BSCB remains under-explored. In this study, we constructed ECs conditional UTX knockout mice and found that UTX knockout significantly improved BSCB disruption and maintained TJs integrity after SCI, as demonstrated through permeability assays and TJs expression assays.

Post-BSCB damage, peripheral immune cells such as macrophages infiltrate the damaged spinal cord tissue. Blood-derived macrophages begin to appear three days after injury, peaking at around 7 days [38]. These infiltrating macrophages contribute to the injury microenvironment by providing protein hydrolases, ROS, and inflammatory cytokines [53, 54]. Their actions can significantly alter blood–brain barrier (BBB)-related gene expression in patients, mediating neuroinflammation [55]. Additionally, perivascular macrophages can exacerbate neurovascular dysfunction by producing large amounts of ROS [56]. In the current study, we found that knocking out UTX in ECs reduced BSCB destruction, significantly reduced macrophage infiltration, and lowered ROS levels in areas of SCI. These findings confirm the role of UTX deletion in vascular stabilization and attenuation of inflammatory responses after SCI.

To elucidate the molecular mechanisms through which UTX regulates BSCB permeability after SCI, we conducted an RNA-seq analysis, which revealed a significant downregulation of MLCK in the ECs with UTX-specific ablation. MLCK is a critical regulator of the vascular barrier’s response to mechanical and inflammatory stimuli, as well as endothelial cell permeability [57, 58]. It is a calmodulin (CaM)-dependent kinase that phosphorylates MLC to cause ATP-dependent actin contraction, cytoskeletal contraction, cytosolic retraction, and TJs detachment. ZO-1 stabilization at TJs is also associated with MLCK-dependent regulation of vascular barrier function [39]. Previous studies have demonstrated that ZO-1 stabilization at TJs is related to the regulation of vascular barrier function. This regulation in vivo needs the action of MLCK, indicating that MLCK-dependent ZO-1 exchange is crucial for barrier regulation mechanisms [59]. Tinsley et al. added MLCK into the medium of monolayer coronary vein ECs and found that it significantly increased the level of MLC phosphorylation (~ 60%) and was accompanied by an increase in the amount of albumin across the endothelium. The other two previous studies showed that inhibition of MLCK using MLCK inhibitors ML-9 and ML-7 decreased endothelial cell permeability and improved blood–brain barrier permeability, respectively [60, 61]. Our study using ChIP-qPCR confirmed that the regulatory complex responsible for altered BSCB permeability after SCI includes UTX and MLCK. Our findings suggest that specific knockout in ECs suppresses actin contraction by inhibiting the MLCK/p-MLC pathway, reducing cytoarchitectural retraction, and preserving TJ protein integrity, thereby reducing BSCB permeability and promoting neurological recovery after SCI.

In conclusion, our study establishes a significant correlation between the spatial and temporal patterns of BSCB permeability alteration after SCI and the expression trend of UTX in the SCI tissue. Our findings confirm the crucial role of UTX in regulating vascular stability and reducing BSCB permeability. Mechanistically, UTX epistemically controls the MLCK/p-MLC pathway after SCI to maintain TJs integrity and BSCB permeability, thereby reducing inflammatory cell infiltration and ROS levels and promoting functional recovery. Importantly, a deeper understanding of UTX's role in BSCB restoration offers a fresh approach and theoretical foundation for the treatment of SCI.

Schematic diagram of the mechanism by which UTX regulates BSCB permeability and inflammatory infiltration after SCI via the MLCK/p-MLC pathway. After SCI, UTX is increased in SCMECs and forms an epistatic regulatory complex with histone H3K27 and MLCK. This complex phosphorylates MLC, leading to ATP-dependent actin contraction, cytoskeleton contraction, cytosolic retraction, the separation of the TJs proteins Claudin-5 and Occludin, and structural changes in ZO-1. These changes ultimately cause structural disruption and increased permeability of BSCB, inflammatory cell infiltration and elevated ROS levels. When UTX was conditionally knocked out in SCMECs, these processes were suppressed, reducing BSCB permeability, limiting inflammatory infiltration and ROS levels, and enhancing mice neurological recovery after SCI.

### Supplementary Information

**Additional file 1: Figure S1. a** Representative fluorescence images of spinal cord specimens from EB leakage experiments at different time points before and after SCI in WT mice. Scale bar, 500 μm. **b**, **c** Quantitative evaluation of EB leakage area and fluorescence intensity in (**a**). Each group *n*=3. **d** Western blotting analysis of the TJs-related protein levels including ZO-1, Occludin, and Claudin-5 in the sham and SCI 3d groups of WT mice. **e** Quantitative analysis of the expression levels of ZO-1, Occludin, and Claudin-5 in (**d**). *n*=3 per group. **f** qRT-PCR verification of the mRNA levels of Claudin-5, Occludin, and ZO-1 in the sham and SCI 3d groups of WT mice. *n*=3 per group. Data are represented as mean ± SEM. *P<0.05, **P<0.01. **Figure S2. **The TJs structure of SCMECs was disrupted after OGD.** a** Representative morphological images of SCMECs. Scale bar, 20 μm. **b** Representative immunofluorescence images of CD31^+^ ECs (green) and DAPI (blue) staining. Scale bar, 10 μm. **c** Representative flow cytometric Histogram of SCMECs with surface marker CD31. **d** Schematic diagram of Transwell FITC-dextran permeation assay. upper layer, vascular endothelial cells. **e**, **f** FITC-dextran transports assay the permeability of WT SCMECs at pre- and post-OGD. *n*=3 per group. **g** Representative immunofluorescence images of TJs-related protein (Claudin-5, Occludin, and ZO-1) in the WT SCMECs when exposed to OGD. Scale bar, 20μm. **h** Quantitative evaluation of the fluorescence intensity of Claudin-5, Occludin and ZO-1 in (**g**). *n*=6 per group. **i** Quantitative evaluation of intercellular distance in (**g**). *n*=6 per group. **j** qRT-PCR verification of the mRNA levels of Claudin-5, Occludin, and ZO-1 in the WT SCMECs when exposed to OGD. Data are represented as mean ± SEM. ns *P*＞0.05, **P<0.01. **Figure S3. a** Schematic diagram of transgenic mice breeding.** b** Western Blotting to identify the expression levels of UTX in UTX^f/f^ and UTX^−/−^ ECs. **c** Quantification of the expression level of UTX in (**b**). Each group *n*=3.** d** Representative fluorescence images of spinal cord specimens from EB leakage experiments at different time points before and after SCI in UTX^f/f^ and UTX^−/−^ mice. Scale bar, 500 μm. **e**, **f** Quantitative evaluation of EB leakage area and fluorescence intensity in (**d**). *n*=3 per group. **g** Western blotting analysis of the TJs-related protein levels including ZO-1, Occludin, and Claudin-5 in UTX^f/f^ and UTX^-/-^ mice at sham and SCI 3d. **h** Quantitative analysis of the expression levels of ZO-1, Occludin, and Claudin-5 in (**g**). *n*=3 per group. **I**–**m** qRT-PCR verification of the mRNA levels of Claudin-5, Occludin, and ZO-1 in UTX^f/f^ and UTX^−/−^ mice at sham and SCI 3d. *n*=3 per group. Data are represented as mean ± SEM. ns P＞0.05, *P<0.05, **P<0.01. **Figure S4. a** Representative fluorescent images of SCMECs after successful transfection with lentivirus. Scale bar, 100 μm. **b** Representative fluorescent images of different time points after intrathecal injection of lentivirus into the spinal cord. Scale bar, 500 μm. **c** Quantitative evaluation of GFP fluorescence intensity in (**b**). *n*=3 per group. **d** Representative fluorescence images of spinal cord specimens from EB leakage experiments at SCI 3d in UTX^f/f^, UTX^-/-^, UTX^-/-^+LV CON, and UTX^−/−^+LV MLCK-OE mice. Scale bar, 200 μm. **e**, **f** Quantitative evaluation of EB leakage area and fluorescence intensity in (**d**). *n*=3 per group. Data are represented as mean ± SEM. ns *P*＞0.05, *P<0.05, **P<0.01. **Figure S5. a** Representative phalloidin fluorescence images of SCMECs. Scale bar, 20μm. **b** Quantitative evaluation of mean fluorescence intensity in (**a**). *n*=6 per group. Data are represented as mean ± SEM. ns *P*＞0.05, **P<0.01.

## Data Availability

The datasets used and/or analyzed during the current study are available from the corresponding author on reasonable request.
