# Supplementary material for: Inhibition of UTX/KDM6A improves recovery of spinal cord injury by attenuating BSCB permeability and macrophage infiltration through the MLCK/p-MLC pathway
Source: J Neuroinflammation. 2023 Nov 11;20:259. doi: 10.1186/s12974-023-02936-1 (PMC10638785; doi:10.1186/s12974-023-02936-1)
Supplement: Supplementary file 1 — Additional file 1: Figure S1. a Representative fluorescence images of spinal cord specimens from EB leakage experiments at different time points before and after SCI in WT mice. Scale bar, 500 μm. b, c Quantitative evaluation of EB leakage area and fluorescence intensity in (a). Each group n=3. d Western blotting analysis of the TJs-related protein levels including ZO-1, Occludin, and Claudin-5 in the sham and SCI 3d groups of WT mice. e Quantitative analysis of the expression levels of ZO-1, Occludin, and Claudin-5 in (d). n=3 per group. f qRT-PCR verification of the mRNA levels of Claudin-5, Occludin, and ZO-1 in the sham and SCI 3d groups of WT mice. n=3 per group. Data are represented as mean ± SEM. *P<0.05, **P<0.01. Figure S2. The TJs structure of SCMECs was disrupted after OGD. a Representative morphological images of SCMECs. Scale bar, 20 μm. b Representative immunofluorescence images of CD31+ ECs (green) and DAPI (blue) staining. Scale bar, 10 μm. c Representative flow cytometric Histogram of SCMECs with surface marker CD31. d Schematic diagram of Transwell FITC-dextran permeation assay. upper layer, vascular endothelial cells. e, f FITC-dextran transports assay the permeability of WT SCMECs at pre- and post-OGD. n=3 per group. g Representative immunofluorescence images of TJs-related protein (Claudin-5, Occludin, and ZO-1) in the WT SCMECs when exposed to OGD. Scale bar, 20μm. h Quantitative evaluation of the fluorescence intensity of Claudin-5, Occludin and ZO-1 in (g). n=6 per group. i Quantitative evaluation of intercellular distance in (g). n=6 per group. j qRT-PCR verification of the mRNA levels of Claudin-5, Occludin, and ZO-1 in the WT SCMECs when exposed to OGD. Data are represented as mean ± SEM. ns P＞0.05, **P<0.01. Figure S3. a Schematic diagram of transgenic mice breeding. b Western Blotting to identify the expression levels of UTX in UTXf/f and UTX−/− ECs. c Quantification of the expression level of UTX in (b). Each group n=3. d Representative [file 12974_2023_2936_MOESM1_ESM.docx]

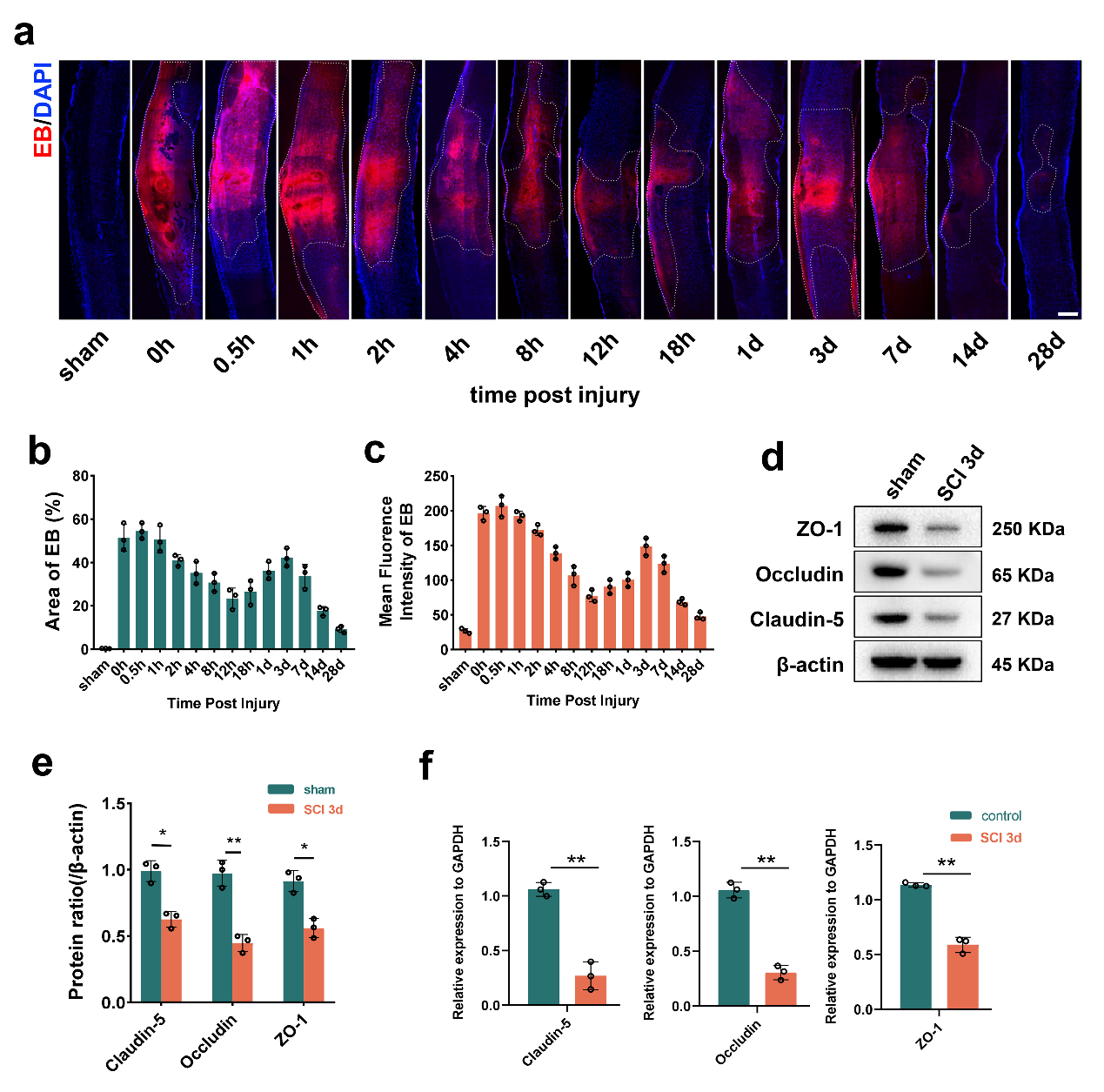


**Additional file 1: Figure S1.** (a) Representative fluorescence images of spinal cord specimens from EB leakage experiments at different time points before and after SCI in WT mice. Scale bar, 500 μm. (b-c) Quantitative evaluation of EB leakage area and fluorescence intensity in (a). Each group n=3. (d) Western blotting analysis of the TJs-related protein levels including ZO-1, Occludin, and Claudin-5 in the sham and SCI 3d groups of WT mice. (e) Quantitative analysis of the expression levels of ZO-1, Occludin, and Claudin-5 in (d). n=3 per group. (f) qRT-PCR verification of the mRNA levels of Claudin-5, Occludin, and ZO-1 in the sham and SCI 3d groups of WT mice. n=3 per group. Data are represented as mean ± SEM. * P<0.05, ** P<0.01.

**
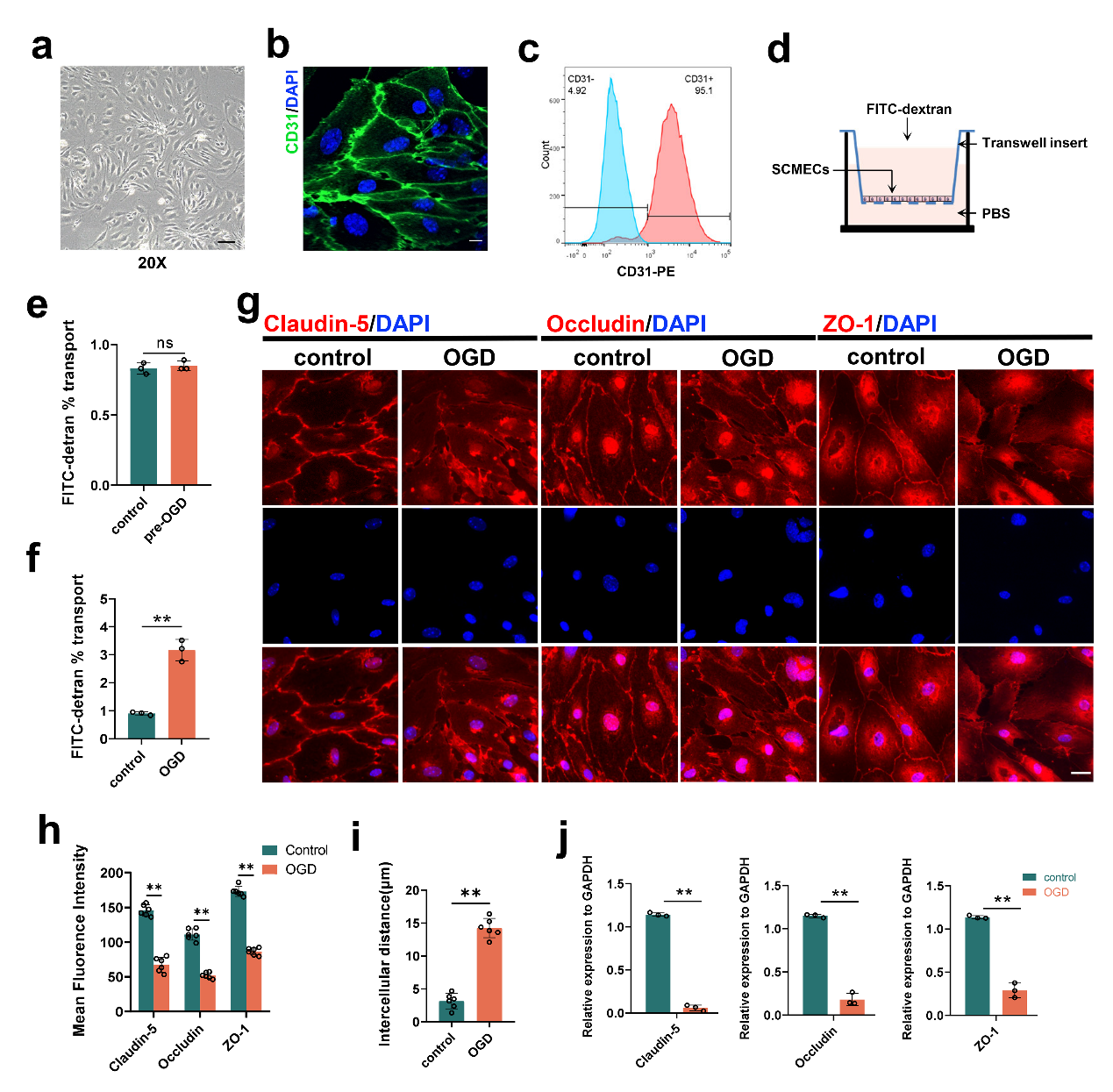
**

**Additional file 1: Figure S2. The TJs structure of SCMECs was disrupted after OGD.** (a) Representative morphological images of SCMECs. Scale bar, 20 μm. (b) Representative immunofluorescence images of CD31^+^ ECs (green) and DAPI (blue) staining. Scale bar, 10 μm. (c) Representative flow cytometric Histogram of SCMECs with surface marker CD31. (d) Schematic diagram of Transwell FITC-dextran permeation assay. upper layer, vascular endothelial cells. (e-f) FITC-dextran transports assay the permeability of WT SCMECs at pre- and post-OGD. n=3 per group. (g) Representative immunofluorescence images of TJs-related protein (Claudin-5, Occludin, and ZO-1) in the WT SCMECs when exposed to OGD. Scale bar, 20μm. (h) Quantitative evaluation of the fluorescence intensity of Claudin-5, Occludin and ZO-1 in (g). n=6 per group. (i) Quantitative evaluation of intercellular distance in (g). n=6 per group. (j) qRT-PCR verification of the mRNA levels of Claudin-5, Occludin, and ZO-1 in the WT SCMECs when exposed to OGD. Data are represented as mean ± SEM. ns P＞0.05, ** P<0.01.


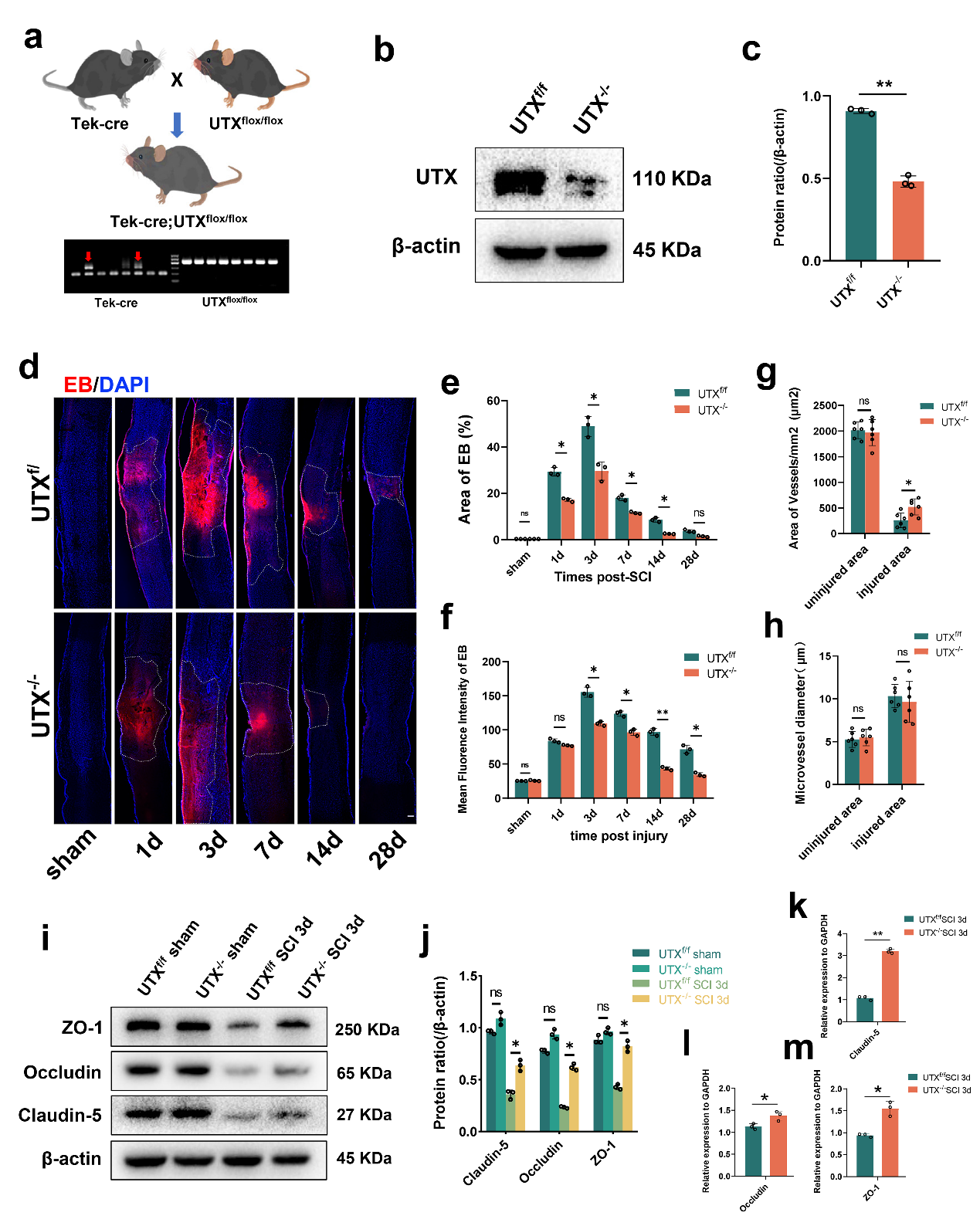


**Additional file 1: Figure S3.** (a) Schematic diagram of transgenic mice breeding. (b) Western Blotting to identify the expression levels of UTX in UTX^f/f^ and UTX^-/-^ ECs. (c) Quantification of the expression level of UTX in (b). Each group n=3. (d) Representative fluorescence images of spinal cord specimens from EB leakage experiments at different time points before and after SCI in UTX^f/f^ and UTX^-/-^ mice. Scale bar, 500 μm. (e-f) Quantitative evaluation of EB leakage area and fluorescence intensity in (d). n=3 per group. (g) Western blotting analysis of the TJs-related protein levels including ZO-1, Occludin, and Claudin-5 in UTX^f/f^ and UTX^-/-^ mice at sham and SCI 3d. (h) Quantitative analysis of the expression levels of ZO-1, Occludin, and Claudin-5 in (g). n=3 per group. (i-m) qRT-PCR verification of the mRNA levels of Claudin-5, Occludin, and ZO-1 in UTX^f/f^ and UTX^-/-^ mice at sham and SCI 3d. n=3 per group. Data are represented as mean ± SEM. ns P＞0.05, * P<0.05, ** P<0.01.


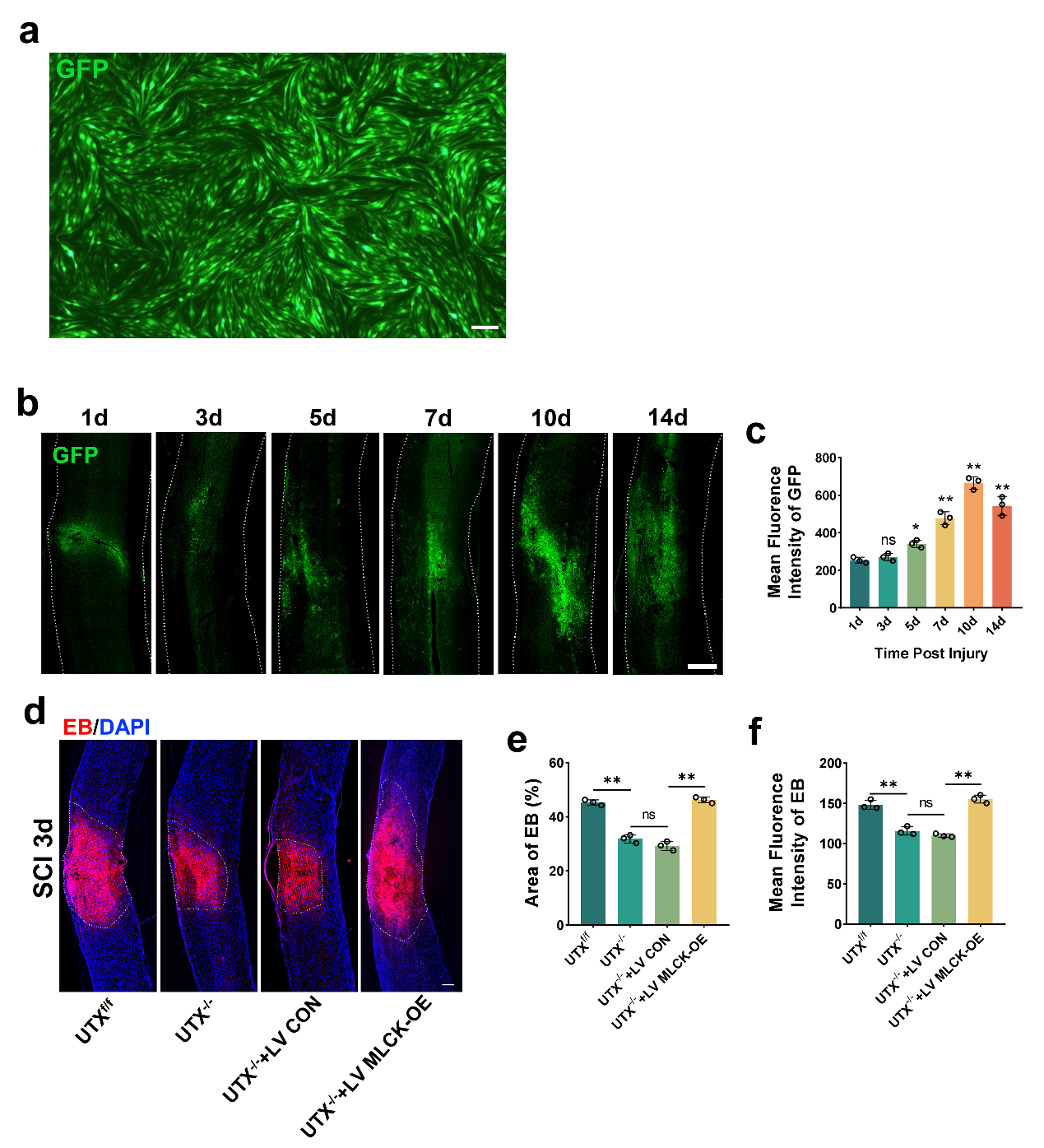


**Additional file 1: Figure S4.** (a) Representative fluorescent images of SCMECs after successful transfection with lentivirus. Scale bar, 100 μm. (b) Representative fluorescent images of different time points after intrathecal injection of lentivirus into the spinal cord. Scale bar, 500 μm. (c) Quantitative evaluation of GFP fluorescence intensity in (b). n=3 per group. (d) Representative fluorescence images of spinal cord specimens from EB leakage experiments at SCI 3d in UTX^f/f^, UTX^-/-^, UTX^-/-^+LV CON, and UTX^-/-^+LV MLCK-OE mice. Scale bar, 200 μm. (e-f) Quantitative evaluation of EB leakage area and fluorescence intensity in (d). n=3 per group. Data are represented as mean ± SEM. ns P＞0.05, * P<0.05, ** P<0.01.


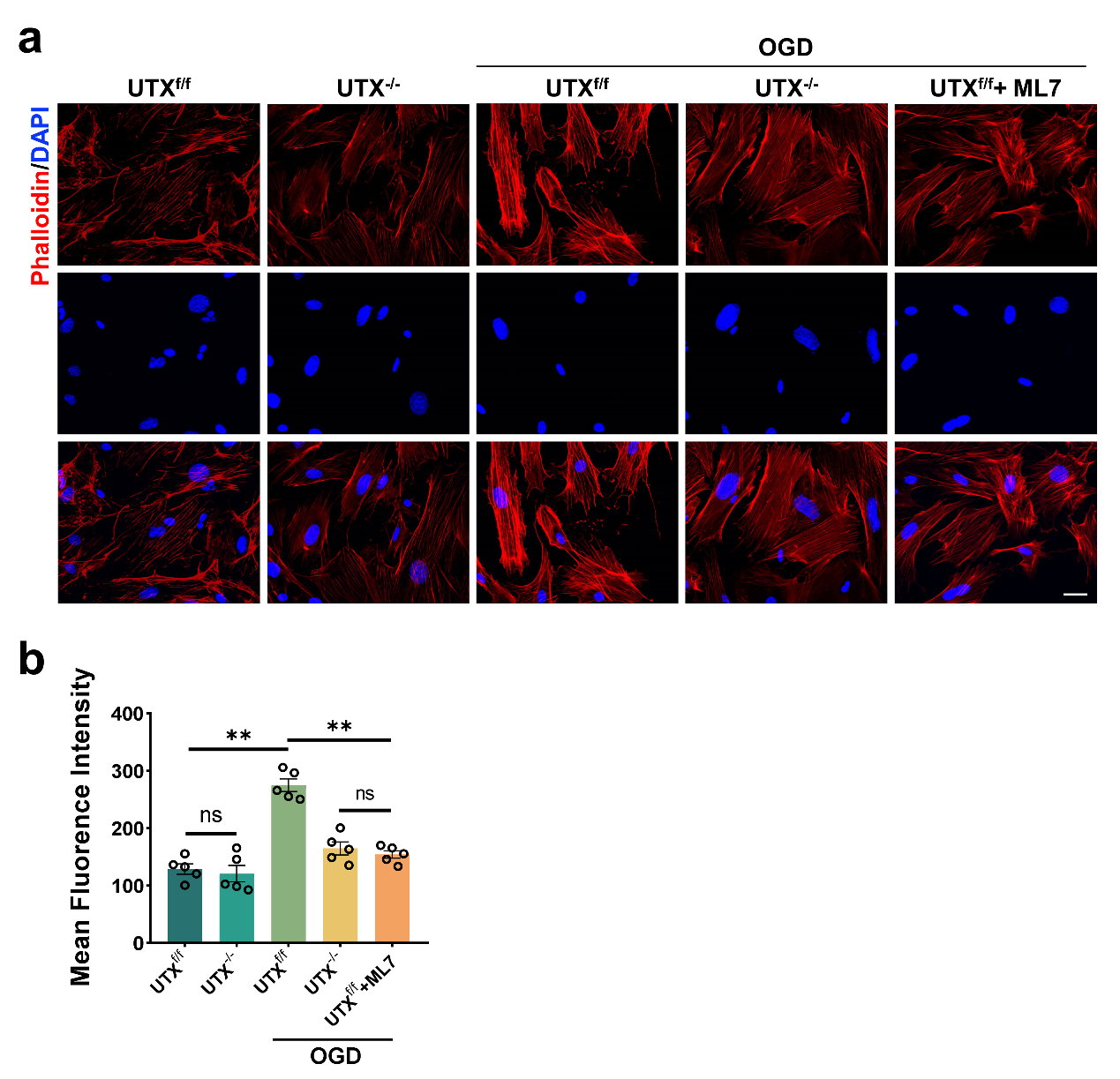


**Additional file 1: Figure S5.** (a) Representative phalloidin fluorescence images of SCMECs. Scale bar, 20μm. (b) Quantitative evaluation of mean fluorescence intensity in (a). n=6 per group. Data are represented as mean ± SEM. ns P＞0.05, ** P<0.01.
